# Anti-Oxidative, Anti-Inflammatory and Anti-Apoptotic Effects of Flavonols: Targeting Nrf2, NF-κB and p53 Pathways in Neurodegeneration

**DOI:** 10.3390/antiox10101628

**Published:** 2021-10-15

**Authors:** Maja Jazvinšćak Jembrek, Nada Oršolić, Lucija Mandić, Anja Sadžak, Suzana Šegota

**Affiliations:** 1 Division of Molecular Medicine, Ruđer Bošković Institute, 10000 Zagreb, Croatia; 2 School of Medicine, Catholic University of Croatia, 10000 Zagreb, Croatia; 3 Department of Animal Physiology, Faculty of Science, University of Zagreb, 10000 Zagreb, Croatia; norsolic@yahoo.com; 4 Division of Physical Chemistry, Ruđer Bošković Institute, 10000 Zagreb, Croatia; Lucija.Mandic@irb.hr (L.M.); Anja.Sadzak@irb.hr (A.S.); Suzana.Segota@irb.hr (S.Š.)

**Keywords:** flavonols, neuroprotection, p53, Nrf2, NF-κB, oxidative stress, neuroinflammation, neurodegeneration

## Abstract

Neurodegenerative diseases are one of the leading causes of disability and death worldwide. Intracellular transduction pathways that end in the activation of specific transcription factors are highly implicated in the onset and progression of pathological changes related to neurodegeneration, of which those related to oxidative stress (OS) and neuroinflammation are particularly important. Here, we provide a brief overview of the key concepts related to OS- and neuroinflammation-mediated neuropathological changes in neurodegeneration, together with the role of transcription factors nuclear factor erythroid 2–related factor 2 (Nrf2) and nuclear factor-κB (NF-κB). This review is focused on the transcription factor p53 that coordinates the cellular response to diverse genotoxic stimuli, determining neuronal death or survival. As current pharmacological options in the treatment of neurodegenerative disease are only symptomatic, many research efforts are aimed at uncovering efficient disease-modifying agents. Natural polyphenolic compounds demonstrate powerful anti-oxidative, anti-inflammatory and anti-apoptotic effects, partially acting as modulators of signaling pathways. Herein, we review the current understanding of the therapeutic potential and limitations of flavonols in neuroprotection, with emphasis on their anti-oxidative, anti-inflammatory and anti-apoptotic effects along the Nrf2, NF-κB and p53 pathways. A better understanding of cellular and molecular mechanisms of their action may pave the way toward new treatments.

## 1. Introduction

Neurodegenerative diseases represent a growing health care, social and economic threat nowadays [1]. They are characterized by progressive neuronal dysfunction and ultimately neuronal loss, leading to cognitive, emotional and behavioral changes that profoundly impair the quality of life. At the molecular and cellular levels, diverse intertwined mechanisms contribute to the progressive nature of these disorders. Together with the unique fingerprint that is typical for each of these diseases (mostly related to distinct proteins that form deposits and the selective vulnerability of specific brain areas), they all have several underlying mechanisms in common. These include oxidative stress (OS), impairment of mitochondrial function, excitotoxicity, chronic inflammatory response and gliosis, protein misfolding, aggregation and accumulation, deregulation of autophagy and proteosomal protein degradation, alterations in the brain lipid profile and disturbed ceramide metabolism, impairment of synaptic function and, ultimately, neuronal death [2,3,4,5,6,7,8].

Particularly concerning are Alzheimer’s disease (AD) and Parkinson’s disease (PD) due to their high prevalence in the elderly population. Briefly, AD is characterized by severe impairment of cognitive abilities, inappropriate emotional and social behaviors and personality changes. The defining clinicopathological hallmarks of AD are deposits of amyloid precursor protein (APP)-derived amyloid-β peptides (Aβ) in the brain parenchyma, and intracellular aggregates of truncated and hyperphosphorylated tau protein in neurofibrillary tangles. Aβ readily self-assembles into diffusible oligomers, the most dangerous form of Aβ species capable of inhibiting synaptic function and plasticity [9,10,11,12]. Tau is a microtubule-associated protein indispensable for microtubule assembly and stability, and axon dynamics. If hyperphosphorylated, tau detaches from microtubules, disturbing tau–microtubule interactions, cytoskeletal arrangement, axonal transport and overall neuronal functioning [9,13]. PD is the most prevalent neurodegenerative disease with prevailing movement deficits. Increased OS, mitochondrial dysfunction and dyshomeostasis of α-synuclein are pivotal mechanisms underlying progressive neurodegeneration in PD [14,15,16]. In physiological conditions, α-synuclein is abundantly expressed in presynaptic neuronal terminals. In PD, it is aggregated, forming intraneuronal inclusions called Lewy bodies and Lewy neurites, which are, together with the specific loss of dopaminergic neurons in the substantia nigra pars compacta, the most important histopathological hallmarks of PD [17,18].

## 2. Oxidative Stress in Neurodegenerative Diseases: The Role of Nrf2 Pathway

OS is one of the most important contributing mechanisms of cellular damage in neurodegenerative diseases [2,6,19,20,21,22]. It arises when the production of reactive oxygen and nitrogen species (ROS and RNS) surpasses endogenous systems of the enzymatic and non-enzymatic anti-oxidative defenses, disturbing cellular redox homeostasis. In AD, OS represents an early event that largely precedes the onset of the first clinical symptoms [23,24,25]. As highly reactive moieties, ROS and RNS react with nearby molecular targets (proteins, DNA, lipids, carbohydrates), disrupting their structure and jeopardizing their function. Besides disturbing their function, oxidative damage of proteins induces their conformational changes and promotes aggregation. Subsequently, protein accumulation leads to widespread microglial activation and triggers the activation of inflammatory pathways and release of pro-inflammatory mediators, which, in turn, further increases ROS generation, OS, protein aggregation and neuronal damage. In this vicious cycle, degradation of misfolded proteins and damaged organelles by the ubiquitin/proteasome system and autophagy is also impaired [5,6,8,10,26,27,28,29,30]. Regarding oxidative damage of lipid molecules, increased production of 4-hydroxy-2-nonenal (HNE), an end product of lipid peroxidation, prevents removal of glutamate by inhibiting glutamate transporters, which together with the ATP depletion, promotes glutamate-mediated excitotoxicity and increases nitric oxide (NO) production. OS also affects the metabolism of lipid signaling molecules and promotes their neurotoxicity [2,31].

In physiological conditions, mitochondria are the major source of ROS production in the electron transport chain of oxidative phosphorylation. In general, mitochondria are highly susceptible to OS-induced damage. When damaged, mitochondria produce escalating amounts of ROS, ultimately leading to ATP deficiency and bioenergetics collapse [3,15,32,33]. Many aspects of mitochondrial dysfunction have been observed in neurodegenerative conditions: change in their size and number, abnormal mitochondrial dynamics, reduced energy metabolism, reduced expression and activity of key enzymes of oxidative metabolism, increased rate of mutations in mitochondrial DNA (mtDNA), increased vulnerability to ROS attack and activation of intrinsic apoptotic pathway components (although not necessarily leading to apoptosis) [7,21,34,35]. OS-induced endoplasmic reticulum (ER) stress, calcium overload and disturbance of mitochondrial calcium homeostasis further contribute to mitochondrial failure and disease progression [7,36,37,38].

In AD, OS and Aβ are reciprocally related. OS induces the production of Aβ, whereas Aβ itself acts as a pro-oxidative agent [10,22,39]. Thus, apart from affecting synaptic transmission, the toxicity of Aβ oligomers is associated with their ability to induce ROS production and promote OS [40]. Furthermore, in the hippocampus and entorhinal cortex of AD patients, reactive astrocytes surrounding Aβ plaques have increased expression of neuronal nitric oxide synthase (nNOS), and neuronal DNA fragmentation roughly matches nNOS expression in astrocytes, indicating a causative role of NO-mediated oxidative damage in AD pathogenesis and interplay between OS and histopathological hallmarks of AD [41]. Moreover, accumulation of Aβ in the mitochondrial matrix contributes to mitochondrial failure and the further enhancement of ROS production [42]. On the other hand, altered mitochondrial function may modulate APP processing and affect the production of amyloidogenic derivatives [43].

Deregulation of metal homeostasis is recognized as one of the pivotal factors contributing to OS in neurodegeneration. Transient metals such as copper, iron and zinc, when present in excess, may catalyze the production of ROS in a Fenton-type reaction, promoting OS induction and exacerbating impairment of neuronal functioning [44,45,46,47]. When bound to Aβ, redox-active metal ions, such as copper, directly promote the production of superoxide anions, hydrogen peroxide and particularly dangerous hydroxyl radicals [21]. Copper coordination to Aβ via His imidazole rings produces hydroxyl radicals close to Tyr residues, leading to tyrosine cross-linking and formation of covalently linked Aβ dimers [48]. This indicates that metal ions, apart from inducing OS, may promote oligomerization of Aβ and increase its neurotoxicity. Similarly, deregulation of copper ions may promote tau hyperphosphorylation and formation of tau fibrils, whereas toxic tau species, in turn, stimulate OS conditions [22,46]. Additionally, OS is accompanied by increased activity of many kinases involved in tau phosphorylation, such as glycogen synthase kinase-3β (GSK-3β), cyclin-dependent kinase 5 (cdk5) and mitogen-activated protein kinases (MAPKs) JNK and p38, and reduced activity of tau-related phosphatases, particularly protein phosphatase 2A (PP2A). Ultimately, these OS-related changes in enzyme activity end in hyperphosphorylation of tau and impairment of tau function [9,46,49,50].

**Figure 1:**
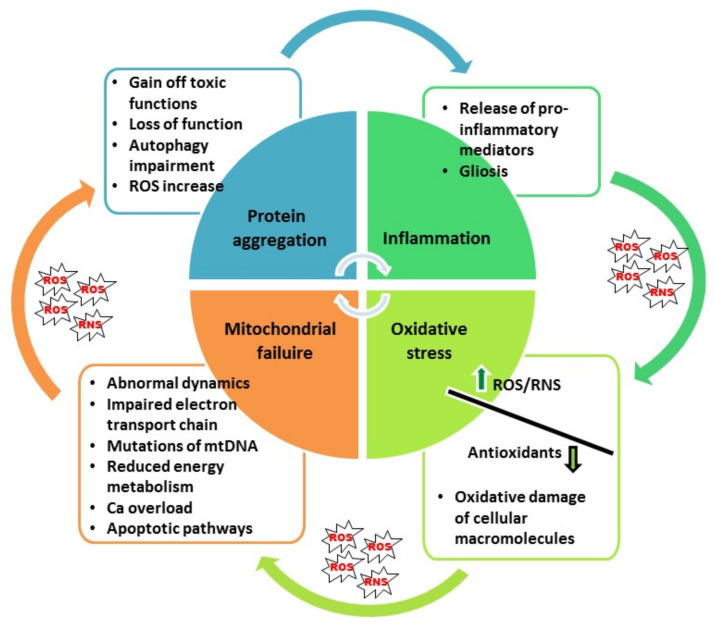
Vicious cycle of oxidative stress, mitochondrial failure, protein aggregation and inflammation in neurodegenerative diseases.

Regarding PD, ATP depletion and ROS-induced oxidative damage, both related to impairment of mitochondrial function, stimulate oligomerization of α-synuclein [14]. As the ubiquitin/proteasome system heavily relies on ATP synthesis, clearance of aggregated proteins is also impeded, further aggravating neuronal functioning [16]. In a transgenic mouse model of PD with overexpression of mutant A30P human α-synuclein and haplodeficiency for superoxide dismutase (SOD) 2, a SOD isoform localized within the mitochondrial matrix, a more advanced synucleinopathy was observed, demonstrating that a reduced capacity for ROS scavenging exacerbates aggregation of α-synuclein [16]. Reversely, accumulation of α-synuclein may induce mitochondrial deficits, increasing OS and leading to neuronal death [51]. Genes involved in inherited forms of PD, such as PTEN induced putative kinase 1 (PINK1) and Parkin, are important for mitochondrial quality control (mitochondrial fission and fusion, and Parkin-related mitophagy), emphasizing a pivotal role of mitochondrial dysfunction and OS in PD [15,18]. Taken together, all these findings demonstrate a causal link between OS, mitochondrial failure and protein aggregation, and their important contribution to neuroinflammation and the further progression of pathological changes and neuronal death (Figure 1).

In response to OS, transcription factor Nrf2 regulates the expression of antioxidants and phase II detoxifying enzymes involved in anti-oxidative defense and is considered as the master regulator of redox homeostasis. Its targets are heme oxygenase-1 (HO-1), SOD1, catalase, glutamate cysteine ligase, glutathione S-transferase and glutathione peroxidase (GSH-Px), among others. Besides this anti-oxidative role, Nrf2 ameliorates apoptotic and inflammatory pathways as well [52,53,54]. The transcriptional activity of Nrf2 is negatively regulated by the repressor protein Keap1 that promotes degradation of Nrf2. In OS conditions, interactions between Keap1 and Nrf2 are disrupted, and Nrf2 translocates to the nucleus where it interacts with antioxidant response element (ARE) and drives the transcription of antioxidant genes [53]. Nrf2 is particularly important for the maintenance of mitochondrial integrity. Hence, it is considered that activation of Nrf2 is likely to be beneficial for the preservation of mitochondrial function and integrity in OS conditions [55].

## 3. Neuroinflammation in Neurodegenerative Diseases: The Role of NF-κB Pathway

Neuroinflammation indicates an immune response of the brain [56]. Prolonged and unregulated inflammation and reactive gliosis have been recognized as a self-perpetuating process in neurodegeneration that causes additional damage to the brain tissue. Neuroinflammation exacerbates impairment of neuronal functioning and speeds up synaptic and neuronal loss, development of symptoms and progression of disease. Chronic inflammation is driven by the activation and proliferation of resident microglial cells, the major component of the innate immune response in the brain, and, to a lesser extent, by reactive astrogliosis [5,6,27,29,56]. In general, the inflammatory response in neurodegenerative diseases should represent a protective mechanism aimed to reduce further damage of the brain tissue. Microglial cells should be essential for maintaining brain homeostasis by removing cellular debris and initiating tissue repair, but overactivation of microglial cells initiates an unregulated pro-inflammatory response that promotes tissue injury [29,57]. Under pathophysiological conditions, activated microglia may differentiate into a continuum of states between phenotypically polarized M1 and M2 states. M1 microglia, whose polarization prevails in neurodegenerative diseases, exert neurotoxic effects and produce pro-inflammatory cytokines and chemokines that maintain inflammatory processes, whereas M2 microglia are neuroprotective, produce anti-inflammatory cytokines and terminate inflammation, contributing to tissue repair [58,59]. The most important pro-inflammatory and cytotoxic mediators produced by M1 microglial cells are cytokines tumor necrosis factor (TNF)-α, interleukin (IL)-1β, IL-1 and IL-6, and inhibition of their production may be an important factor for reducing neuronal damage. Anti-inflammatory molecules produced by M2 microglia are IL-10, tumor growth factor β (TGFβ) and neurotrophic factors [60].

The major activators of the immune response in neurodegenerative diseases are danger-associated molecular patterns (DAMPs) originating from the damaged tissue, including aggregated proteins and various molecules originating from damaged or dying neurons [61,62]. These endogenous motifs are recognized by pattern recognition receptors (PRRs), which include a wide spectrum of receptors such as Toll-like receptors, the nucleotide-binding oligomerization domain leucine rich repeat-containing receptors and scavenger receptors, among others [6,63,64,65].

Activation of PRRs transduces the signal to the nucleus and activates transcription factor NF-κB that drives the production of pro-inflammatory cytokines and chemokines, prostaglandins, free radicals such as NO and superoxide anions, and activation of the inflammasome, ultimately ending in chronic neuroinflammation [58,66]. NF-κB represents a family of inducible transcription factors composed of five members: NF-κB1 (p50), NF-κB2 (p52), RelA (p65), RelB and c-Rel. In the form of homo- and hetero-dimers, these transcription factors bind to specific DNA elements named κB enhancers and mediate transcription of target genes [58]. NF-κB proteins are sequestered in the cytoplasm by inhibitory proteins such as IκB family members, of which IκBα is the best characterized [58]. NF-κB transcription factors can be activated by the canonical and non-canonical pathways. In contrast to the non-canonical pathway that represents an alternative option, the canonical pathway regulates almost all types of immune responses. It responds to PRRs and is activated by the degradation of IκBα [58,64]. Increased acetylation of NF-κB p65 is an important step for the activation of NF-κB. NAD^+^-dependent histone deacetylase sirtuin 1 (silent mating type information regulation 2 homolog 1, Sirt1) acts on NF-κB and decreases acetylation at Lys310, thus attenuating the transcriptional activity of NF-κB and pro-inflammatory processes [67].

After activation of NF-κB, the produced cytokines and chemokines recruit resident microglial cells to the site of damage. Activated microglia, in turn, produce ROS and promote OS. NF-κB also drives the expression of NAPDH oxidase (NPHOX) that produces superoxide anions. As oxidative damage of mitochondria reduces the production of endogenous ATP and rises the concentration of glutamate, excitotoxic processes result in the activation of inducible nitric oxide synthase (iNOS) and generation of NO. NO in combination with superoxide anions forms the extremely dangerous peroxynitrite, a powerful oxidizing and nitrating species that is considered as one of the major causes of neuronal injury in neurodegeneration [38,68,69,70].

## 4. The Role of p53 in Neurodegenerative Diseases

Besides activation of the Nrf2 and NF-κB pathways, other intracellular signaling pathways help in orchestrating the neuronal response to injury. These include mitogen-activated protein kinases (MAPKs), i.e., pathways of kinase p38, extracellular kinase (ERK1/2) and JNK kinase, and the p53 pathway, among others [10,57,60,71]. Long-lasting activation of JNK and p38 by OS and pro-inflammatory cytokines largely contributes to neuronal death in neurodegenerative diseases. It has been shown that p38 mediates activation of p53 which then induces Bax expression and promotes permeabilization of the outer mitochondrial membrane. Activated p38 may also induce expression of iNOS and increase the production of NO [72].

**Figure 2:**
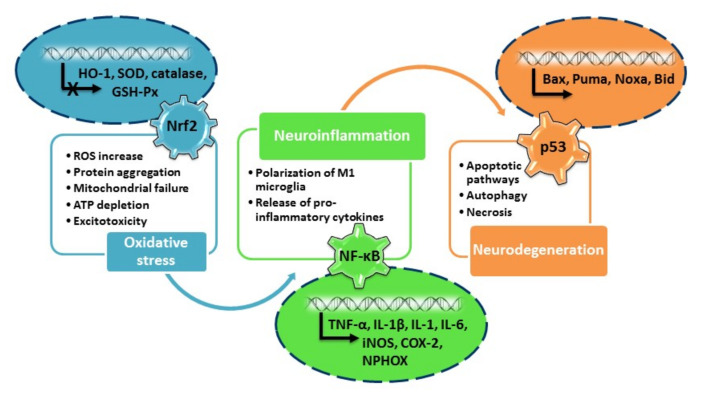
The involvement of transcription factors Nrf2, NF-κB and p53 in oxidative stress, neuroinflammation and neurodegeneration.

In general, intracellular signaling pathways form an interconnected network important from the various aspects of neuronal functioning (Figure 2). For example, signaling pathways are highly implicated in neuroprotective activities of the DJ-1 protein in PD. Mutations of the *PARK7* gene coding for DJ-1 account for early onset cases of PD. DJ-1 acts as an anti-oxidative agent, molecular chaperone and transcriptional co-activator, optimizes a number of mitochondrial functions and protects mitochondria against OS. In OS conditions, DJ-1 regulates the Nrf2, NF-ҡB, p53 and Akt pathways, contributing to activation of anti-oxidative and metabolic neuroprotective mechanisms, and inhibition of phosphatase activity, ultimately regulating the preservation of the redox balance and offering protection against α-synuclein-mediated neurodegeneration [73].

As the roles of the Nrf2, NF-κB and MAPK pathways in anti-oxidative and neuroinflammatory responses have been described in detail by others [54,55,64,74,75,76], we review the role of the p53 pathway in the development and progression of neurodegenerative diseases and discuss its contribution to OS- and neuroinflammatory-related events in detail.

### 4.1. Biological Functions and Structure of p53

The transcription factor p53 is a redox-regulated protein with multiple cellular functions, nowadays receiving growing attention in the field of neurodegenerative diseases [10,57,77,78]. Acting as a transcriptional regulator, it guides the cellular response to various endogenous and exogenous stress stimuli, particularly those that induce genotoxic effects, thus having a prominent role in DNA repair and maintenance of genome stability. It determines cell fate by promoting either cell proliferation or cell death, controls different metabolic functions and energy metabolism (participates in the regulation of glucose levels, ATP production, glycolysis, oxidative phosphorylation and glutamine and lipid metabolism), shapes anti-oxidative responses and regulates inflammation [57,79,80,81,82,83]. Hence, impairment or aberrant activation of p53 activity may threaten neuronal functioning and result in pathological outcomes, including those related to neurodegenerative diseases [10,78,84,85,86].

Regarding protein structure, 12 distinct p53 isoforms have been identified—p53α/β/γ, Δ40p53α/β/γ, Δ133p53α/β/γ, and Δ160p53α/β/γ. Briefly, by using two different promoters, by initiating translation from two sites within the P1 promoter and by alternative splicing at intron 2 or intron 9, transcription of the TP53 gene gives rise to four major isoforms: full-length p53, and three truncated forms, ∆40p53, ∆133p53 and ∆160p53, shortened at the N-terminus. Additional α, β and γ variants are formed through alternative splicing [87,88,89,90,91]. The functional significance of isoform diversity is not understood, particularly in the brain. In general, p53α (canonical p53) associates with the transcriptionally active tetrameric complex regulating the transcription of over 3000 genes [89]. These downstream targets of p53 are cellular mediators of its function. Considering neurodegenerative diseases, of particular interest are p53-regulated genes involved in apoptosis, such as Bax, p53-upregulated mediator of apoptosis (PUMA) and Noxa, and genes involved in cellular responses to OS and regulation of other redox-sensitive signaling pathways [91,92]. Proteins p63 and p73, homologues of p53, together with p53 comprise the p53 family and contribute to a complex signaling network that orchestrates the expression of specific patterns of proteins, regulating the survival or apoptosis of neuronal cells during injury [93,94,95,96].

### 4.2. Regulation of p53 Activity

In physiological conditions, in the absence of stressful stimuli, p53 is a very unstable and inactive protein, continuously degraded by the proteasome. The stability and transcriptional activity of p53 are mainly regulated by several protein–protein interactions and posttranslational modifications, including phosphorylation, acetylation, ubiquitination, methylation and sumoylation, among others. Stabilization of p53 in the cytoplasm promotes its translocation into the nucleus and activation of transcriptional activity, whereas specific posttranslational modifications may affect its specificity of binding to distinct p53-responsive elements, ensuring preferential activation of a specific pool of p53-regulated proteins [57,90,92,95,97,98,99].

The murine double minute-2 (Mdm2) protein is one of the principle negative regulators of p53 activity and is also its downstream target. It acts as an E3 ubiquitin ligase, labeling p53 for proteasome-dependent degradation, and inhibiting its nuclear import by ubiquitination [100,101]. Posttranslational modifications that are introduced after DNA damage, of which phosphorylation and acetylation are particularly relevant, interrupt p53/Mdm2 interactions and promote p53 oligomerization, nuclear accumulation and increases in activity [99,102,103]. Another important regulator of p53 function is Mdm4 (Mdmx). It binds Mdm2, and the hetero-dimer formed promotes ubiquitination of p53 by Mdm2 and p53 degradation. However, under severe DNA damaging conditions, Mdm4 dissociates from Mdm2, cooperates with p53 and promotes its activity. Mdm4 also stimulates phosphorylation of p53 at Ser46 by binding to and stabilizing homeodomain interacting protein kinase 2 (HIPK2). Ultimately, phosphorylation by HIPK2 promotes the transcriptional repression of targets with anti-apoptotic activity [104].

**Figure 3:**
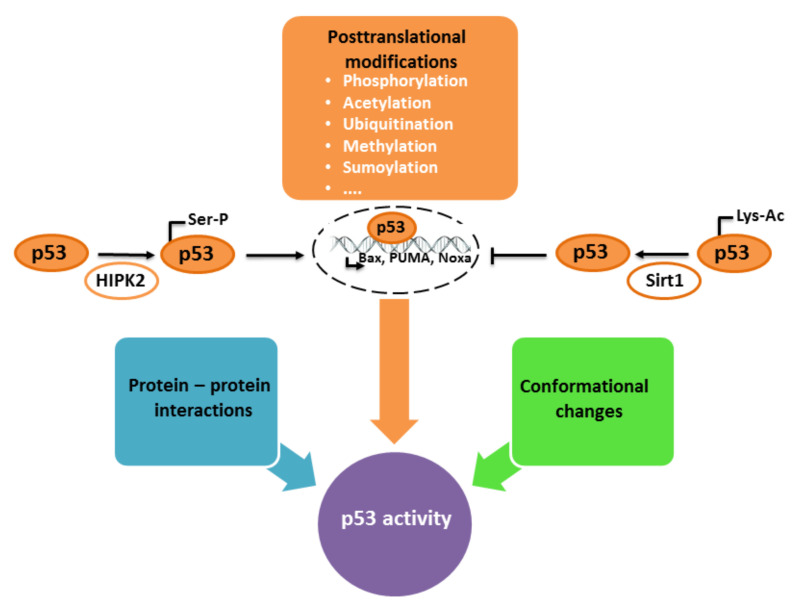
Regulation of p53 activity in physiological and neurodegenerative conditions.

Furthermore, the effects of p53 are partially mediated by sirtuins, a family of proteins with histone deacetylase activity. Sirtuins play an important role during cellular responses to various stressors, including oxidative and genotoxic stress [105]. p53 is a deacetylation target of Sirt1. Acetylation of p53 increases transactivation and promotes the expression of various p53-regulated genes. However, acetylation competes with the ubiquitination, sumoylation and methylation of lysine residues, thus enabling the fine-tuning of p53-guided cellular responses to various challenges [106]. Increased acetylation of p53 has been observed in neuronal responses to various noxious stimuli [107,108], whereas inhibition of deacetylase activity may protect neuronal cells against apoptotic death, partially by suppressing p53-dependent expression of PUMA, activation of Bax and initiation of the caspase-3 enzymatic apoptotic cascade [109,110] (Figure 3).

To a lesser extent, the activity of p53 is affected by its conformational state. In peripheral cells of AD patients, p53 is conformationally altered, conferring higher resistance to genotoxic stimuli. It has been revealed that very small concentrations of Aβ40 may contribute to inappropriate p53 folding [84,111]. Low amounts of Aβ downregulate levels of HIPK2 and induce impairment of HIPK2 binding to DNA. This Aβ-mediated deregulation of HIPK2 is likely responsible for the observed p53 misfolding, suppression of p53 transcriptional activity and aberrant neuronal vulnerability [111]. Levels of molecular chaperones Hsp70 and Hsp90 may also contribute to the regulation of the DNA binding activity of p53. Hsp70 unfolds and inactivates p53, but when p53 is transferred from Hsp70 to Hsp90, it restores its native conformation and activity [112].

Finally, subtle modifications of p53 activity may be governed by the cellular redox status and availability of zinc ions. Although ROS and oxidative damage of DNA are important triggers for p53 activation, p53 is susceptible to oxidative environments which may change its conformation and activity. Redox-inert zinc is essential for p53 activity as the core domain of p53 binds one zinc atom. Zinc offers protection from oxidation and is important for DNA binding. However, if present in excess, zinc induces structural changes of p53 and inhibits its binding to DNA. Copper, which is per se able to increase the production of ROS, may displace zinc from its binding site on p53. This results in an aberrant protein structure and loss of p53 activity [113,114,115].

### 4.3. Role of p53 in Neuronal Death

The levels and activity of p53 are substantially increased in various acute and chronic neuropathological conditions accompanied with OS and DNA damage. p53 is predominantly involved in the activation of apoptotic signaling pathways and the development of brain damage [116,117]. The best characterized apoptotic p53 function is related to its transcriptional regulatory role. p53 drives the expression of a panel of pro-apoptotic genes, simultaneously suppressing the expression of anti-apoptotic genes from the Bcl-2 family [118]. The principal mediators of pro-apoptotic p53 function in mature cortical neurons are Bax and PUMA. In OS conditions, PUMA is the main regulator of Bax activation, and in cortical neurons, PUMA represents the critical link between p53 and Bax [91]. Bax-mediated permeabilization of the mitochondrial membrane triggers the release of pro-apoptotic effector cytochrome c, facilitating the formation of an apoptosomal complex and the initiation of the apoptotic caspase cascade [119,120]. The pivotal role of p53, Bax and PUMA in neuronal death has been suggested in various in vitro and in vivo settings [91,121,122,123,124]. Besides regulating the expression of genes from the Bcl-2 family, p53 regulates the expression of some microRNAs (miRNAs), of which those from the miR-34 family are the most prevalent. Expression of miR-34a, which targets Bcl-2 transcripts for degradation, increases with age in the mouse and rat brain and might contribute to a p53-related increased rate of neuronal death [77,120,125].

In non-neuronal cells, p53 may exert its effects via a transcription-independent mechanism. This mode of action is based on the accumulation of p53 in the cytoplasm and mitochondria, and specific protein–protein interactions. In this transcription-independent mode of action, p53 behaves similarly to a BH3-only protein. By directly interacting with anti-apoptotic and pro-apoptotic Bcl-2 family members, p53 regulates the permeabilization of the outer mitochondrial membrane and initiates the apoptotic cascade. HIPK2-mediated phosphorylation induces cis/trans isomerization of cytoplasmic p53 that results in p53 mono-ubiquitination, promoting its translocation to mitochondria. In mitochondria, p53 undergoes rapid de-ubiquitination, binds to resident Mdm4 on the outer membrane and interacts with anti-apoptotic proteins Bcl-2 and Bcl-xL, inhibiting their function and inducing membrane permeabilization. Acetylation of p53 at Ser120 further contributes to oligomerization of Bak, a pro-apoptotic member of the Bcl-2 family, which is, together with Bax, important for the permeabilization of the mitochondrial outer membrane [118,126]. Although there is not enough evidence for a transcription-independent p53 action in neuronal cells, a p53 translocation to mitochondria has been observed in human SH-SY5Y neuroblastoma cells exposed to chlorpyrifos, an acetylcholinesterase inhibitor that induces OS, suggesting that, in some circumstances, mitochondrial p53 activity could be involved in neuronal apoptosis [127].

In addition to apoptosis, p53 may regulate autophagy [128]. Autophagy is required for the elimination of damaged proteins and mitochondria which is particularly important in brains affected by neurodegenerative diseases [129]. Autophagy (including mitophagy) requires transcriptional and non-transcriptional p53 action. In general, nuclear p53 promotes autophagy by transactivating an array of autophagy-related genes, whereas the non-transcriptional effects of cytoplasmic p53 are evident in basal conditions and they are inhibitory [130]. However, the transcriptional activity of p53 may also downregulate autophagy. It has been shown that nuclear p53 could repress the promoter activity of PINK1, a key regulator of mitophagy. Negative regulation of autophagy by p53 is PINK1 dependent, which might indicate that inhibition of autophagy/mitophagy by nuclear p53 may contribute to pathological events in neurodegenerative diseases [128]. In normal brain tissue, the essential autophagy gene *Atg7* acts as a negative regulator of p53 and limits its activation [129]. Furthermore, in the rat striatum exposed to traumatic brain injury (TBI), expression of p53 was co-localized with microtubule-associated light chain (LC3), an autophagy marker [131]. In yet another study, in delayed neurodegeneration after TBI, nuclear expression and activity of p53 were found to be upregulated in the cortex and hippocampus and correlated with posttraumatic neurodegeneration in injured brain tissue, whereas the transcriptional activity of NF-ҡB was reduced. As inhibition of p53 reduced neurodegeneration and upregulated the levels of NF-ҡB protein targets, this suggests a coordinated response of p53 and NF-ҡB in neuronal injury [132]. Moreover, it has been shown that overstimulation of N-methyl-D-aspartate (NMDA) receptors (which are involved in excitotoxicity) induces NF-ҡB-dependent expression of p53 and activates neuronal death via p53-dependent apoptotic and autophagic mechanisms [122,133].

Regarding the role of p53 in the regulation of OS-induced necrosis, p53 may activate necrotic death after the bioenergetic collapse of the cell by opening the mitochondrial permeability transition pore (mPTP), which results in the dissipation of the mitochondrial membrane potential [134].

Accordingly, mounting evidence indicates that suppression of p53-mediated activities by genetic or pharmacological manipulations may attenuate deleterious effects of various noxious stimuli, reduce neuronal damage and promote neuronal survival [131,132,133,135,136,137]. Pifithrin-α is a widely used inhibitor of transcriptional p53 activity that positively modulates neuronal viability in various experimental settings. It rescued neuronal cultures against glutamate, Aβ and DNA-damaging agents by reducing p53 activation, expression of Bax and PUMA, mitochondrial failure and caspase-3 activity [119,137,138]. In TBI-induced damage of striatal neurons, pifithrin-α reduced not only OS, apoptosis and autophagy but also neuroinflammation, again indicating mutual connections between intracellular signaling pathways [131]. Similarly, neurons of mice administered pifithrin-α were less vulnerable to excitotoxic damage [137,139], and animals subjected to mild TBI and pifithrin-α were more resistant to later cognitive deficits [138].

### 4.4. Role of p53 in Neurodegenerative Diseases

Enhanced p53 expression, as well as oxidatively modified forms of p53, has been observed in cortical neurons and glia of transgenic AD mice and post-mortem human AD brains, in a good correlation with mitochondrial and other dysfunctions [77,140,141,142,143]. Oxidative DNA damage triggers nuclear localization of Aβ42, whereas Aβ42 directly activates the *TP53* promoter and induces p53-dependent apoptosis [141]. In addition, p53 is capable of indirectly stimulating tau phosphorylation [9,142] or may interact with tau oligomers [144]. It has been shown that the Δ40p53 isoform, whose level increases during aging, promotes tau phosphorylation by upregulating the transcription of several tau kinases [145]. In primary neuronal cultures, it has been shown that exposure to oligomerized Aβ25-35 promotes phosphorylation of p53, its stabilization and induction of p53-responsive genes Bax and PUMA, leading to mitochondrial depolarization and impairment of mitochondrial function and neuronal apoptosis. Treatment with Aβ25-35 also activates cdk5 which, in turn, phosphorylates p53. Accordingly, pharmacological inhibition of cdk5 or p53 prevents mitochondrial dysfunction and neuronal death. The physiological relevance of these findings has been confirmed in the mouse brain where Aβ25-35 promoted p53 accumulation, dendrite disruption and neuronal apoptosis in the hippocampus and cortex, clearly demonstrating the important role of p53 in determining susceptibility to Aβ toxicity, and the potential of targeting p53 in AD [10,146].

Interestingly, p53 oligomers and fibrils are also detected in AD brains. In response to DNA damage, p53 is normally phosphorylated. In AD brains, phosphorylated p53 forms oligomers which are localized outside the nucleus, probably underlying the lack of a robust p53 response and delayed neuronal apoptosis. In accordance with this, despite the evidence of increased DNA damage in AD brains, levels of acetylated p53 and DNA damage responders transcriptionally regulated by p53 are found to be reduced, suggesting a breakdown in the p53-mediated DNA damage response pathway, probably due to p53 aggregation and cytoplasmic sequestration, or possibly interactions with tau oligomers [144]. Another study demonstrated elevated levels of p53 monomers and dimers in AD brains, together with selective glutathionylation of these forms. The authors suggested that glutathionylation may affect the formation of tetrameric complexes that are required for the optimal interaction of p53 with DNA, which potentially might have some implications for the pathogenesis of AD [143].

Regarding PD, injection of 1-methyl-4-phenyl-1,2,3,6-tetrahydropyridine (MPTP) induces an increase in ROS, and DNA damage, and activates nuclear enzyme poly(ADP-ribose)polymerase (PARP). p53 is heavily poly(ADP-ribosyl)ated by PARP-1 following MPTP treatment. As p53 stability and activity are mainly regulated by posttranslational modifications, poly(ADP-ribosyl)ation increases transactivation of p53 target genes and presumably serves as a regulator of MPTP-induced neuronal apoptosis [97]. In addition, it has been shown that MPTP treatment induces selective activation of the p38 kinase in dopaminergic neurons, phosphorylation of p53 and its nuclear translocation and transactivation of Bax and PUMA [147]. As for AD, a reciprocal regulatory control exists between p53 and PD-specific proteins. For example, p53 is a substrate of leucine-rich repeat kinase 2 (LRRK2) whose mutation causes PD. Phosphorylation of p53 by LRRK2 induces apoptosis of differentiated SH-SY5Y cells and rat primary neurons [148].

Expression of p53 has also been investigated in astrocytes. In general, interactions between astrocytes and neurons can be neurotoxic (mediated by a senescence-associated secretory phenotype and production of pro-inflammatory cytokines) or neuroprotective (attributed to the release of neurotrophic growth factors). The differential expression of isoforms Δ133p53 and p53β is important for shaping the overall effect of astrocytes on neuronal cells. In particular, downregulation of Δ133p53 or upregulation of p53β correlates with the neurotoxic functions. Of note, reduced expression of the Δ133p53 isoform that promotes neuroprotective effects, and upregulation of the p53β isoform that stimulates toxic effects of astrocytes are found in the brain tissue of AD patients [149].

Finally, it has to be mentioned that p53 may coordinate protective molecular mechanisms rather than promoting the death of postmitotic neurons. These findings could indicate an alternative explanation for the increased expression of p53 in the diseased brain. Thus, in a *Drosophila* model of tau pathology, transcription of genes controlling synaptic function and protection from tau neurotoxicity was linked with p53-mediated neuroprotective functions [150]. In addition, it is known that acetylation of p53 at Lys320 is important for neurite outgrowth and maturation in vitro, and physiological nerve regeneration in vivo, in part by transactivating the expression of the actin-binding protein Coronin 1b and the small GTPase Rab13, which have multiple cellular functions, including the dynamic remodeling of the cytoskeleton [151,152]. Moreover, transcription of a prototypical axon growth factor, growth-associated protein 43 (GAP-43), which contributes to axon sprouting and regeneration, is also regulated by p53 [153]. Finally, it has been shown that the Mdm2/p53 pathway is important for brain preconditioning-induced neuroprotection. Brain preconditioning indicates a state of transient resistance against severe ischemic damage evoked by a prior non-injurious exposure to NMDA. Preconditioning increases Mdm2 levels, thus preventing ischemia-induced activation of the p53/PUMA/caspase-3 signaling cascade [154].

#### 4.4.1. p53 Functions in Oxidative Stress

Intracellular metabolism of ROS is regulated by p53, at least partially. When OS levels are low, p53 promotes antioxidant activities and, ultimately, survival. If OS levels exceed the coping abilities of neuronal cells, p53 drives a pro-oxidative response, leading to death. Although the switch mechanism underlying the polarity of the p53 reaction is not understood, it is accomplished through the activation of a distinct subset of genes and pathways involved in the OS response [92,155].

Persistent activation of p53, which is characteristic of neurodegenerative diseases, increases the production of ROS. Firstly, p53 upregulates the expression of ROS, producing pro-oxidant genes, including p53-inducible gene 3 (*PIG3*, coding for quinone oxidoreductase), proline oxidase gene (*PRODH*), *p66shc*, *Bax* and *PUMA*. Pro-apoptotic targets BAX and PUMA disturb mitochondrial function through the release of pro-apoptotic mediators, indirectly resulting in upregulation of ROS production due to the reduced efficiency of the electron transport chain. Secondly, p53 represses the expression of anti-oxidative genes, also leading to an increase in ROS. In particular, p53 may repress the transcription of the *SOD2* gene (coding for mitochondrial or manganese superoxide dismutase) and several phase II antioxidant response genes including those for glutathione-S-transferase, NAD(P)H quinone oxidoreductase and the cystine-glutamate transporter [91,155]. Interestingly, p53 also inhibits the expression of *SOD2* and antioxidant response genes in physiological conditions, suggesting that low p53 activity in healthy cortical neurons is predominantly pro-oxidant. In pathological conditions, when p53 is activated, the redox balance is further pushed toward a more pro-oxidant state [91]. Consistently, attenuation of p53 activity decreases basal levels of OS in the mouse brain [156]. In comparison with wild-type animals, the protein carbonyl content, 3-nitrotyrosine and levels of HNE were reduced in p53 knock-out mice, together with a downregulated nuclear fraction of Nrf2 and increased levels of NF-κB, thioredoxin-1 and SOD2 [156]. Hence, therapeutic strategies focused on the inhibition of pro-oxidative p53 activity could be a promising approach in attenuating OS, neuroinflammation and progression of other OS-mediated neurodegenerative changes.

##### p53 and Mitochondrial Functions in Neurodegenerative Conditions

From the aspect of OS, aging and neurodegeneration, mitochondrial p53 functions have received increased attention [130,157]. p53 regulates mitochondrial functions at many levels. It has been shown that p53 controls mitophagy, a specific form of autophagy that eliminates damaged, ROS-producing mitochondria. Mitophagy is vital for sustaining redox homeostasis and neuronal survival under OS. However, when the levels of OS are high, mitophagy is decreased, which results in the accumulation of damaged mitochondria and massive cell death [8]. In OS conditions, mitophagy is regulated by the PINK1–Parkin axis. PINK1 accumulates at the depolarized mitochondria and is required for Parkin recruitment to impaired mitochondria and activation of its kinase activity. Phosphorylation of Parkin activates its E3 ubiquitin ligase activity, and it ubiquitylates numerous outer mitochondrial membrane proteins, labeling damaged mitochondria for autophagic removal. In non-neuronal tissues, cytosolic p53 can bind to Parkin. This prevents its mitochondrial translocation and inhibits ubiquitin ligase activity, interfering with Parkin’s biological function in mitochondrial quality control, and limiting its ability to promote mitophagy [158,159,160]. On the other hand, nuclear p53 controls the expression of PINK1 by repressing its transcription, thus downregulating mitophagy which may be relevant for the impairment of mitochondrial function, ROS production and neurodegeneration [128]. Besides PINK1 that is critically involved in autophagy/mitophagy, mitochondrial proteins involved in glycolysis and mitochondrial respiration, and other aspects of mitochondrial metabolism, are also controlled by p53, indicating a contribution of p53 in coupling energy metabolism and ROS formation [82,155].

Recently, it has been shown that exposure of a whole cell to DNA damage activates p53-dependent apoptotic degeneration in the cell bodies, but p53-independent axonal degeneration after selective mtDNA damage [161]. In fact, some transcriptional targets of p53 are involved in the maintenance of mtDNA. Moreover, following translocation into mitochondria, p53 helps in maintaining the genomic stability of mtDNA by interacting with mtDNA and proteins involved in the maintenance of mtDNA [130]. Furthermore, in AD, structural damage, an increase in size and a decrease in the mitochondrial number are observed [162]. The appropriate number and shape of mitochondria are highly important for neuronal cells due to their high energy demands [163]. Proteins involved in mitochondrial fusion/fission are also controlled by p53. Thus, dynamin-related protein 1 (Drp1), a main regulator of mitochondrial fission (a process that is important for the initiation of apoptosis), is transcriptionally activated by p53 [164]. Drp1 binds to p53 and is required for p53 translocation to the mitochondria in acute OS-induced necrosis related to brain ischemia [165] and in vitro and in vivo models of Huntington’s disease (HD) [166]. Similarly, pharmacological inhibition of Drp1 hyperactivation prevents the death of dopaminergic neurons by inhibiting p53-mediated apoptosis, likely by preventing Drp1-regulated translocation of p53 to mitochondria [164]. In addition, p53 may promote Drp1-dependent mitochondrial fission by suppressing the transcription of miR-499 that ultimately reduces the accumulation of Drp1 in mitochondria [167].

Dysfunction of mitochondrial homeostasis is considered particularly important for PD and HD. Several mitochondrial toxins (such as rotenone, paraquat and MPTP) that directly or indirectly damage mitochondria are routinely used to mimic pathological changes characteristic of PD [168]. These drugs enhance the production of ROS, induce oxidative damage and trigger OS-related apoptotic cascades. Many lines of evidence indicate that α-synuclein induces the dysfunction and death of dopaminergic neurons by accumulating in mitochondria. Overexpression of mitochondrial α-synuclein enhances the production of ROS, reduces mitochondrial bioenergetics and the ATP content and induces pronounced structural damage of the mitochondria and mitochondrial fragmentation, depolarization of the mitochondrial membrane, severe degeneration of the neurite network and neuronal death [169,170,171]. Interestingly, α-synuclein also reduces the expression and transcriptional activity of p53 [172]. On the other hand, pharmacological and genetic manipulations that increase p53 expression also increase the levels of the α-synuclein protein, its promoter activity and mRNA levels, indicating that α-synuclein is a p53 target [173]. Furthermore, p53 was found to be heavily poly(ADP-ribosyl)ated after exposure to MPTP. After entrance, MPTP is converted to MPP^+^ that acts as a complex I inhibitor. Inhibition of complex I upregulates ROS production, induces DNA damage and activates PARP-1. PARP poly(ADP-ribosyl)ation stabilizes p53 and influences its transcriptional activity as it prevents binding of p53 to DNA [97].

#### 4.4.2. p53 and Neuroinflammation

The immune response in the brain could be modulated by p53. In general, p53 regulates the expression of effector genes which promote the inflammatory response (inflammatory phenotype of microglial cells) and is usually not activated in the anti-inflammatory M2 phenotype [57,174]. Hence, prevention of p53 activation could be a promising target for attenuating destructive neuroinflammation in neurodegenerative diseases. Interestingly, a common polymorphism at codon 72 in the *TP53* gene that encodes either proline or arginine influences the immune activities of p53, at least in the thymus. Following ionizing radiation, the Pro72 variant induced more prominent apoptosis, together with increased transactivation of p53 target genes. Most of the transactivated genes were related to inflammation and contained NF-κB binding sites, ensuring an enhanced response to inflammatory challenges in comparison with mice having the Arg72 variant [79].

Concomitantly with p53 activation and microglial secretion of pro-inflammatory cytokines TNF-α and IL-1β, a significant decrease in the expression of synaptic markers has been observed in neuronal cells, before any visible signs of neuronal death, suggesting that p53 activities in microglia may affect synapse integrity and exacerbate synapse deterioration [175]. Accordingly, microglia from p53-deficient mice do not secrete pro-inflammatory cytokines and fail to upregulate other pro-inflammatory genes but show increased expression of genes that mediate anti-inflammatory responses, phagocytosis and regenerative functions [57,174]. It has been revealed that activation of p53 induces the expression of specific miRNAs, promoting pro-inflammatory and inhibiting anti-inflammatory behavior [57]. Thus, p53 influences microglial behavior by regulating the anti-inflammatory transcription factor c-Maf. Two p53-regulated microRNAs, miR-34a and miR-145, negatively regulate Twist2, a transcriptional activator of c-Maf, as well as c-Maf itself. Similarly, microglia from p53-deficient mice have increased expression c-Maf, clearly indicating the important influence of p53 activation on microglial functions [176]. Another mechanism by which p53 could influence the immune response is by regulating the expression of Toll-like receptors as the majority of family members have p53 response elements [177].

**Figure 4:**
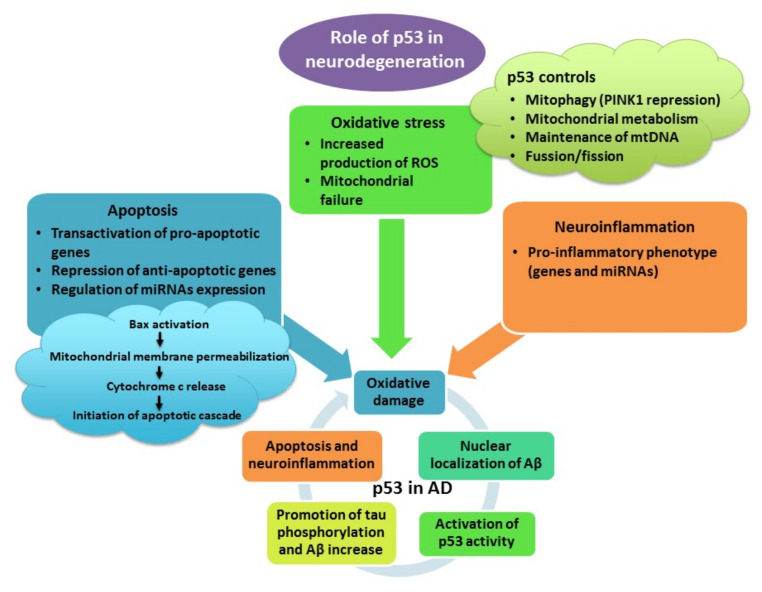
The contribution of p53 to neuronal death, oxidative stress and neuroinflammation. The mutual connections between p53, Aβ and tau in Alzheimer’s disease are indicated.

It is known that ROS-induced DNA damage upregulates the expression and activity of p53 in microglial cells [174,176]. Not surprisingly, p53 expression can also be induced by the Aβ peptide, which, as mentioned before, may increase the production of ROS. Exposure to Aβ increases p53 immunoreactivity in primary rat microglia, rendering microglial cells more prone to apoptosis. Accordingly, pifithrin-α significantly reduces p53 expression and attenuates microglial apoptosis. As activation of p53 occurs before iNOS induction, a possible link between p53 activation and iNOS transcription has been suggested [178]. Furthermore, as conditioned media from microglia treated with Aβ induce apoptosis of cultured cerebellar granule neurons, the release of soluble neurotoxins following p53 activation was suggested [178]. In another study, expression of p53 and its downstream target PUMA was increased in neurons, astrocytes and microglia following spinal cord ischemia reperfusion. Overexpressed p53 was colocalized with NF-κB, and attenuation of PUMA expression by the small interfering RNA approach, as well as treatment with pifithrin-α, prevented translocation of NF-κB, release of pro-inflammatory cytokines IL-1β and TNF-α and, ultimately, caspase-3-mediated neuronal apoptosis [179]. Similarly, in TBI-induced striatal damage, pifithrin-α suppressed neuroinflammation, glial activation and release of pro-inflammatory cytokines TNF-α, IL-1β and IL-6 and improved functional deficits. However, it also prevented upregulation of HO-1, which is an Nrf2 target, and attenuated lipid peroxidation, altogether demonstrating the pro-oxidative and pro-inflammatory effects of p53 and its partnership with NF-κB and Nrf2 in the neuronal response to injury (Figure 4) [131].

## 5. Flavonols as Therapeutic Option in Neurodegenerative Diseases

Despite the enormous research efforts and major advances in understanding the molecular and cellular mechanisms underlying the development and progression of neurodegenerative disorders, disease-modifying therapies are still missing. Available therapeutic options are limited and offer only temporary relief from symptoms. At present, it is believed that due to the complex pathophysiology of neurodegenerative diseases, potential therapeutic agents must display multi-target activities [180]. In this regard, bioactive compounds of natural origin, particularly those with a polyphenolic backbone, are highly appreciated as potential therapeutics due to their relative safety and multifunctional biological and pharmacological activities that target many cellular events related to neurodegeneration [56,66,123,181,182,183].

Flavonoids are a heterogeneous group of natural phytochemicals produced by secondary plant metabolism. They are widely distributed in plants and products of plant origin, such as tea and wine, and represent the most abundant group of polyphenolic compounds in daily diets [184,185]. Hence, it comes with no surprise that they are one of the most studied dietary supplements from the aspect of their therapeutic potential in neurodegenerative diseases [182,186,187,188,189,190,191,192].

**Figure 5:**
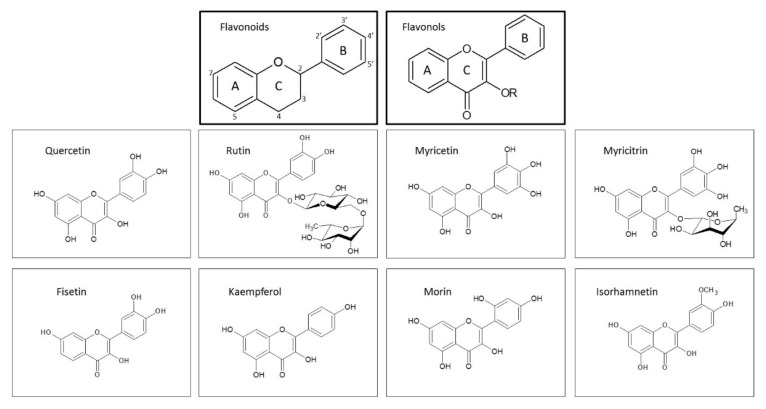
The basic backbone of flavonoids and flavonols, and chemical structures of main flavonols.

All flavonoids possess two benzene rings (rings A and B) that are bridged with a heterocyclic pyrene pyran ring (ring C) (Figure 5). Depending on the position at which the B ring is attached to the C ring, the degree of oxidation and unsaturation of the central ring and the pattern of the attached glycosidic moieties, hydroxyl groups and other substituents, they are classified into several distinct groups. The major groups of flavonoids are flavonols, flavanols, flavones, flavanones, anthocyanins and isoflavones [182,189]. The chemical structure determines the biological activities of different flavonoids, including their anti-oxidative and anti-inflammatory potential [66].

Flavonoids have a long tradition of medical use based on their great diversity and numerous health-promoting effects. Of note, although flavonoids themselves may be used as therapeutic agents, they are important building blocks in drug synthesis. Hence, they are promising neuroprotective candidates from the perspective of medicinal chemistry and drug discovery and development [182,192].

Flavonoids exhibit a plethora of notable biological and pharmacological effects. Their anti-oxidative effects are based on direct ROS-scavenging properties, and the ability to chelate metal ions and positively regulate the expression of enzymatic and non-enzymatic components of the intracellular anti-oxidative defense. By alleviating ROS production, they contribute to the restoration of mitochondrial functions and promote anti-apoptotic and other prosurvival activities, offering protection against various neurotoxins. They increase cerebrovascular functions and blood flow, facilitate angiogenesis, neurogenesis and changes in neuronal morphology, increase the spine density and suppress neuroinflammation and gliosis, altogether exerting beneficial effects on cognitive functioning [181,193]. The anti-inflammatory effects of flavonoids are well documented. They may suppress the activation of microglial cells and prevent the release of ROS/RNS, thus reducing the neurotoxic consequences of oxidative species [56]. Furthermore, they inhibit eicosanoid-generating enzymes (eicosanoids are lipid signaling molecules that regulate inflammatory processes) and modulate the expression of pro-inflammatory genes (e.g., cyclooxygenase-2 (COX-2), iNOS and pro-inflammatory cytokines) [194]. Finally, flavonoids modulate activities along various redox-sensitive signaling cascades involved in OS and inflammatory responses, regulating an intricate network of molecular events underlying neuronal death or survival [123,181,188,193,195].

However, the full potential of flavonoids in neuroprotection is far from closure. It is known that in the presence of transient metals such as copper and iron, flavonoids may act as pro-oxidants and contribute to increases in ROS, intensifying OS and threatening neuronal viability [123,196,197]. Moreover, flavonoids are metabolically altered in the gut after oral consumption. Following uptake and absorption in the small intestine, only small amounts of ingested compounds enter the body and enrich the brain. In the gut, phase 1 metabolism by cytochrome P-450 and phase 2 metabolism by conjugating enzymes liberate water-soluble products, mostly in the form of sulphated and glucuronidated derivatives, whereas remaining fractions are metabolized by the microbiota [195]. All these metabolic alterations affect their bioavailability which, together with the low blood–brain barrier (BBB) penetration, limits their efficacy. Accordingly, many research efforts are directed at improving their availability and biological effects.

Due to the great diversity of flavonoids, herein, we will discuss the effects of flavonoids from the flavonol group.

### 5.1. Quercetin

Quercetin is one of the most studied and pharmacologically best described flavonols. It is highly present in daily diets, predominantly in various fruits, vegetables and beverages. The best sources of quercetin are apples, onions, capers, broccoli, berries and red wine, among others [198]. Many studies have suggested a promising therapeutic potential of quercetin due to its numerous pharmacological and health-beneficial effects. The neuroprotective activities of quercetin have been described in in vitro and in vivo models of neurodegenerative diseases, ischemia and TBI and largely assigned to its anti-oxidative, anti-apoptotic and anti-inflammatory properties [123,186,188,199,200,201]. In addition to its ability to reduce OS and neuroinflammation, quercetin stimulates neuronal regeneration and neurogenesis and enhances the functioning of existing neurons. In general, these effects promote neuronal survival and have positive effects on the preservation of cognitive functions [202].

Quercetin is a very potent antioxidant. Its anti-oxidative effects are achieved through the direct scavenging of ROS moieties, chelation of metal ions and prevention of Fenton chemistry and stimulation of the expression and activity of anti-oxidative enzymes. The anti-oxidative effects of quercetin have been confirmed in numerous in vitro models of oxidative injury. Thus, rat pheochromocytoma (PC12) cells pre-treated with quercetin were protected against H_2_O_2_-induced OS and cell damage. The protective effects of quercetin were assigned to the reduced production of ROS and malondialdehyde (MDA), which is an end product of lipid peroxidation and an OS marker. In PC12 cells, quercetin also ameliorated the activities of anti-oxidative enzymes catalase, SOD and GSH-Px and activated the PI3K/Akt pathway [203,204]. In cerebellar granule neurons exposed to H_2_O_2_, quercetin stimulated nuclear translocation of Nrf2 and increased the expression of its target genes involved in GSH synthesis, with positive effects on neuronal survival [205].

The anti-oxidative effects of quercetin are ultimately reflected in decreased apoptotic rates. Pre-treatment with quercetin preserved nuclear morphology, restored the Bcl-2/Bax ratio (through increased Bcl-2 and reduced Bax expression), suppressed the initiation of caspase signaling and decreased p53 expression [206]. In differentiated P19 neurons, quercetin also promoted survival in a H_2_O_2_-induced oxidative environment. It prevented increases in p53 and downregulation of Bcl-2, at least partially, by modulating the Akt and ERK1/2 signaling cascades [186,188]. Interestingly, the anti-oxidative and neuroprotective effects of quercetin have also been demonstrated in P19 neurons exposed to copper-induced OS, but the levels of the p53 protein were not modulated by exposure to either copper or quercetin [123]. As in copper-induced neuronal death, quercetin was effective in preventing aluminum-induced damage, mostly due to its anti-oxidative and anti-apoptotic properties. In the hippocampus of rats administered with aluminum, pre-treatment with quercetin inhibited ROS generation, restored mitochondrial manganese superoxide dismutase (MnSOD) activity, improved mitochondrial integrity and function, prevented chromatin cleavage by endogenous nucleases, inhibited translocation of cytochrome c from mitochondria to the cytosol and caspase-3 activation, downregulated the expression of p53 and Bax and increased the levels of Bcl-2, ultimately preventing OS-induced neurodegeneration and neuronal death [207]. As copper and aluminum dyshomeostasis is highly implicated in the development of OS in neurodegenerative conditions, it has to be mentioned that in the presence of metal ions, quercetin may exert pro-oxidative effects and exacerbate neuronal death [123]. Hence, current findings demonstrate that the effects of quercetin on p53 induction and neuronal survival are context dependent, which needs to be further clarified in further studies.

A neuroprotective effect of quercetin was also demonstrated in pentylenetetrazol-induced OS in zebrafish. Pentylenetetrazol is a stimulant that triggers ROS production and neuronal apoptosis. The effects of pentylenetetrazol on the reduction in GSH levels and increase in thiobarbituric acid reactive substances (TBARS) in the zebrafish brain were prevented by solid lipid nanoparticles loaded with quercetin. These anti-oxidative activities were accompanied with the restoration of acetylcholinesterase activity and memory improvement [208].

Based on the results of the mentioned studies and many others, the neuroprotective potential of quercetin in AD is highly appreciated. Various desirable effects of quercetin against AD pathology have been confirmed. Quercetin increases the levels of acetylcholine, inhibits the activity of beta-secretase-1 (BACE-1), lowers the production of Aβ, inhibits fibril formation, prevents tau-related pathology and attenuates the proliferation of glial cells, ultimately promoting neuronal survival and improvement in cognitive symptoms [201,209,210]. Inhibition of BACE-1 activity is likely related to an NF-κB-dependent mechanism which, in turn, regulates BACE-1 transcription [211]. Moreover, in PC12 cells exposed to Aβ25-35, quercetin exerted its anti-oxidative effects by increasing the levels of the Sirt1 and Nrf2 proteins, driving the expression of anti-oxidative enzymes [212]. On the other hand, the effects of quercetin on tau pathology are mediated through the downregulation of GSK-3β activity via the enhancement of the PI3K/Akt pathway [213]. In addition, it seems that quercetin conformationally stabilizes tau monomers and prevents the formation of tau fibrils [214]. Similar to the previously mentioned effects in various OS conditions, in vitro models of AD also indicate strong anti-oxidative, anti-apoptotic and prosurvival effects of quercetin. In HT22 cells exposed to okadaic acid, quercetin not only prevented tau hyperphosphorylation and tau pathology but also boosted the anti-oxidative defense by several mechanisms. It reduced the production of ROS, stimulated the activity of SOD2 and GSH-Px, prevented oxidative damage of mitochondrial membrane lipids, enabled preservation of the mitochondrial membrane potential and upregulated intracellular GSH levels [213]. Anti-apoptotic effects were evidenced through the reduced activity of caspase-3 and reversion of the okadaic acid-induced increase in Bax. Quercetin also inhibited the p38 and JNK signaling pathways and activated NF-κB p65 [213]. In another study, quercetin prevented the okadaic acid-induced increase in tau phosphorylation by inhibiting the Ca^2+^-calpain-p25-cdk5 pathway [215]. Together with GSK-3β, cdk5 is considered as one of the major tau kinases in AD [49]. Increased activation of cdk5 is associated with increased apoptosis in neuronal cells. As p53 is a substrate of cdk5, it is likely that cdk5/p25-mediated phosphorylation of p53 facilitates the progression of apoptosis through increased transcriptional activity of p53 and an increase in Bax [216]. This suggests that quercetin-mediated inhibitory effects on p53-driven neurodegenerative processes could be cdk5 related. Of note, by using bioinformatic tools, it is predicted that quercetin may directly interact with p53 and inhibit its activity [202].

More importantly, neuroprotective effects of quercetin administration were also observed in animal models of AD [217]. In APPswe/PS1dE9 transgenic mice, quercetin administered for 16 weeks ameliorated mitochondrial dysfunction, restored the levels of ATP and ROS, reduced plaque pathology and improved learning and memory deficits [209]. In another study performed in 3xTg-AD mice, preventive administration of quercetin for 12 months reduced the aggregation of Aβ and prevented an increase in tau hyperphosphorylation in the CA1 region of the hippocampus and amygdala, with positive effects on cognitive performance and emotional behavior [218].

Apart from AD, beneficial effects of quercetin on the improvement in cognitive abilities were also demonstrated in intact (healthy) animals. Superparamagnetic iron oxide nanoparticles were used as a delivery system to enhance the bioavailability of quercetin in the brain. Ultimately, it has been suggested that the beneficial effects of quercetin interfere with long-term potentiation neurotrophin signaling and apoptotic pathways [202]. In a *Drosophila* model of AD, it was shown that quercetin restores Aβ-induced changes in the expression of genes involved in the OS response and p53 pathway, further indicating the important contribution of p53 signaling in the neuroprotective effects of quercetin [219]. The effects of quercetin on the activation of the Nrf2/ARE pathways were also confirmed in AD animals. Pre-treatment with quercetin stimulated activation of Nrf2 and expression of HO-1 in Aβ-induced AD in rats. Quercetin attenuated lipid peroxidation, increased GSH levels and SOD and catalase activity in brain homogenates and reduced Aβ42 levels and degenerative changes in the hippocampus [220].

In addition to its significant anti-oxidative and anti-apoptotic effects, the anti-inflammatory properties of quercetin are highly appreciated when considering its therapeutic potential in AD [221]. The anti-inflammatory effects of quercetin have been confirmed in numerous studies. Therefore, quercetin inhibits iNOS induction and production of NO, reduces the production of pro-inflammatory cytokines, including TNF-α, IL-1β and IL-6, regulates the expression of COX-2 and suppresses astrocytosis and microgliosis [201,217]. Similarly, in lipopolysaccharide (LPS)-induced neuroinflammation, quercetin reduced the activation of glial cells, attenuated neuroinflammation-related markers (TNF-α, COX-2, IL-1β and iNOS), prevented synaptic dysfunction and neuronal apoptosis in the hippocampus and cortex of adult mice and enhanced memory functions [200]. These effects were accompanied with a decreased expression of TLR-4 receptors and downregulation of p-NF-κB. In the CA1 region of aged 3xTgAD mice, the inhibitory effect of quercetin on the activation of the NF-κB pathway also ameliorated astrogliosis and downregulated inflammatory mediators iNOS, COX-2 and IL-1β [210].

Quercetin has also demonstrated a therapeutic potential in PD. In the rotenone rat model of PD, quercetin restored the brain dopamine level and improved motor deficits, reduced the levels of MDA and activity of thioredoxin reductase, prevented endoplasmic reticulum stress and DNA fragmentation and stimulated autophagy [222]. At least partially, the rotenone-induced apoptosis and induction of p53 expression are mediated by the reduced activity of the protein deacetylase Sirt1. In particular, binding of Sirt1 within the p53 promoter inhibits transcription of the *TP53* gene and thus confers protection against rotenone-induced injury by suppressing p53 expression [223]. Recently, it has been shown that quercetin 3,5,7,3′,4′-pentamethyl ether, a natural sirtuin-activating compound, directly interacts with Sirt1 and strongly stimulates its deacetylase activity by enhancing its binding affinity for p53-derived peptides [224]. Hence, these findings suggest that the effects of quercetin against rotenone-induced toxicity could be mediated via the Sirt1/p53 pathway. Furthermore, in dopaminergic neuronal cells, quercetin applied alone stimulated the Akt signaling pathway, as well as cAMP response element-binding protein (CREB) phosphorylation and brain-derived neurotrophic factor (BDNF) expression, and increased mitochondrial biogenesis and mitochondrial bioenergetics efficiency. These effects are likely involved in the anti-apoptotic and neuroprotective effects of quercetin against 6-hydroxydopamine (6-OHDA)-induced neurotoxicity [225]. More importantly, in the MitoPark transgenic mouse model that recapitulates the course of the disease, oral administration of quercetin together with vitamins C and B3 and folic acid reduced nigrostriatal degeneration, reversed dopamine depletion in the striatum and reduced motor deficits [225], clearly demonstrating the potential of food supplementation in alleviating symptoms of neurodegenerative diseases. In yet another study, the SK-N-MC human neuroblastoma cell line and Sprague Dawley male rats were exposed to excess manganese (Mn), thus inducing changes similar to idiopathic PD. In vitro, pre-treatment with quercetin attenuated Mn-induced OS, increased SOD and catalase activity and the intracellular GSH pool, improved mitochondrial function and prevented apoptosis. At the molecular level, quercetin attenuated NF-κB signaling but activated the Nrf2 pathway. Similar findings were observed in the rat brain. Quercetin reduced the generation of ROS and protein carbonyl levels, increased SOD activity, reduced the expression of neuroinflammatory markers TNF-α, IL-1β, IL-6, COX-2 and iNOS, upregulated the levels of Nrf2 and HO-1 mRNA, depleted NF-κB mRNA and reversed Mn-induced changes in apoptotic markers, including Bax, Bcl-2 and cleaved caspase-3. Together, these anti-oxidative, anti-inflammatory and anti-apoptotic effects of quercetin reduced Mn-induced histopathological changes in the striatum [226].

**Figure 6:**
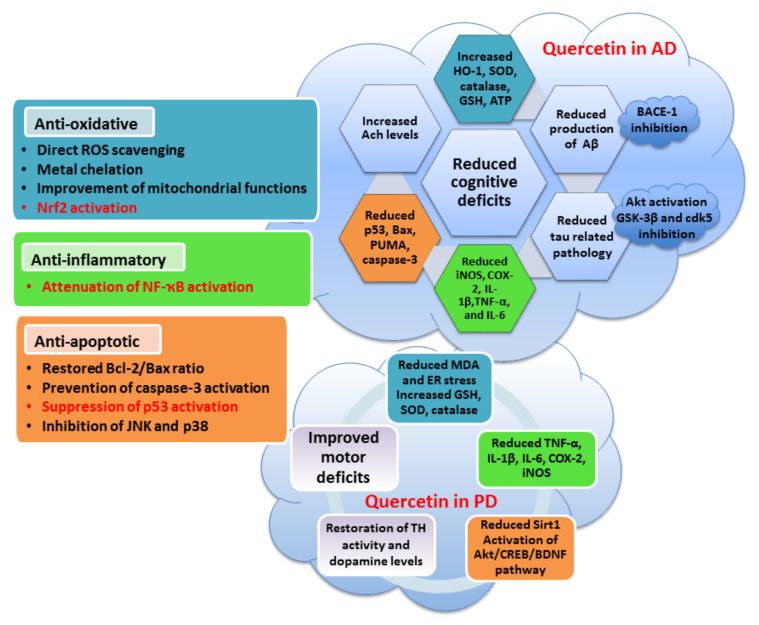
Neuroprotective activities of quercetin in neurodegenerative conditions. Some specific effects of quercetin in Alzheimer’s disease and Parkinson’s disease are indicated.

Beneficial effects of quercetin, mainly on the improvement in mitochondrial functions, were likewise demonstrated in a 3-nitropropionic acid (3-NP)-induced model of Huntington’s disease (HD). In mitochondria originating from the striatum, quercetin preserved the activities of electron transport chain enzymes, maintained ATP levels, attenuated OS and total thiol levels, restored the activities of SOD and catalase and prevented alterations in the mitochondrial membrane structure and function. Restoration of mitochondrial activity was accompanied with the prevention of motor deficits [227]. In another study, quercetin applied together with fish oil also provided neuroprotection and reduced the 3-NP-elicited enhancement of ROS production, as well as levels of MDA, protein carbonyls and NO in the striatum and cerebellum [228]. Upregulation of p53 has been observed in 3-NP-treated animals [229]. In striatal cells, p53 was involved in 3-NP-induced apoptosis and autophagy. In addition to p53, expression of p53 targeted Bax, and PUMA and damage-regulated autophagy modulator (DRAM) were also upregulated [230]. Hence, it is likely that the beneficial effects of quercetin in HD models could be p53 mediated. In conclusion, based on the observed neuroprotective effects of quercetin in various experimental settings, it seems that quercetin possesses great potential for clinical applications in neurodegenerative diseases (Figure 6).

Besides being found in neurodegenerative diseases, neuroprotective effects of quercetin against OS and OS-associated edema have been demonstrated in a rat model of subarachnoid hemorrhage. Treatment with quercetin enhanced the activity of CuZn-SOD and GSH-Px and reduced the MDA content. These anti-oxidative effects attenuated caspase-3 activity in the CA1 region of the hippocampus, reduced brain damage and prevented neurobehavioral deficits [231]. Furthermore, it seems that quercetin could be effective against brain injury of offspring after maternal infection. In Wistar rats, quercetin successfully alleviated fetal brain damage induced by maternal exposure to LPS. In brain tissue of newborn rats, quercetin attenuated the release of pro-inflammatory cytokines IL-1β, IL-6 and TNF-α, suppressed LPS-induced apoptosis by preserving the Bax/Bcl-2 ratio and exerted anti-oxidative effects by reducing MDA levels and by increasing catalase activity and the GSH content [232].

As with other flavonoids, quercetin is characterized by low solubility and absorption, which may limit its biomedical applications. However, despite these limitations, it is still able to cross the BBB, and studies in animals have confirmed its biological effectiveness in medical conditions. Furthermore, delivery of quercetin in the form of nanoparticles, such as superparamagnetic iron oxide nanoparticles [202] and solid lipid nanoparticles [208], may help in overcoming this problem and facilitate its bioavailability and distribution in the brain, thus ensuring better biological efficacy [233].

### 5.2. Rutin

Rutin (quercetin-3-O-rutinoside, vitamin P) possesses an attached sugar rutinose at the C3 position of the quercetin aglycone backbone (Figure 5). As with quercetin, rutin has demonstrated pharmacological properties that could be of interest in the prevention and therapy of neurodegenerative diseases. Supplementation with rutin may prevent neuronal loss in the hippocampus, improve memory impairment and suppress microglial activation and production of pro-inflammatory cytokines [234]. In a rat model of sporadic dementia induced by intracerebroventricular injection of streptozotocin, rutin not only improved cognitive deficits and attenuated inflammation and morphological changes in the CA1 region of hippocampus but also exerted powerful anti-oxidative effects. Its anti-inflammatory action was evidenced by the reduced expression of NF-κB, COX-2 and iNOS, and attenuated glial activation, whereas its anti-oxidative effects were confirmed through reduced TBARS and nitrite levels, an increase in the GSH content and increased activities of anti-oxidative enzymes [235]. Regarding AD, in APP/PS1 and 5XFAD mice, rutin stimulated Aβ phagocytosis and clearance, prevented activation of microglia and astrocytes, reduced the production of inflammatory cytokines, reversed synaptic dysfunction and ameliorated learning and memory deficits [236].

Rutin also protected the rat hippocampus and frontal cortex against oxidative damage related to ischemic stroke. It reduced infarct size, neuronal loss and impairment of motor behavioral performances. At the molecular level, prevention of neuronal death was accompanied with the downregulation of p53 expression. In the same model, rutin preserved the activities of anti-oxidative enzymes, restored the GSH content and attenuated lipid peroxidation and the protein carbonyl content [237]. During oxidative injury, rutin may also attenuate the expression of inflammation markers and levels of NF-κB and suppress p53, ERK1/2 and caspase-3 activity, preventing apoptotic and necrotic death [238].

### 5.3. Myricetin

Myricetin (hydroxyquercetin, 3,3′,4′,5,5′,7-hexahydroxyflavone) is dietary flavonol with a plethora of biological activities. When compared to quercetin, it possesses an additional –OH group at the B ring (Figure 5). It is commonly found in berries, nuts, vegetables, teas and wine [239]. Its polyphenolic backbone is highly hydroxylated (possesses 6 –OH groups), and depending on the environmental conditions, it may act both as an antioxidant and pro-oxidant [240].

Neuroprotective effects of myricetin have been reported, and similar to quercetin, they are mostly assigned to its anti-oxidative and anti-inflammatory effects. Thus, myricetin prevented glutamate-induced Ca^2+^ overload and enhancement of ROS production and suppressed caspase-3 activation by directly interacting with the caspase-3 active site [241]. The therapeutic potential of myricetin in AD and PD is also appreciated. Regarding AD, in primary cortical neurons, myricetin reduced the neurotoxic effects of Aβ, demonstrating anti-amyloidogenic properties. It prevented conformational changes of Aβ and the consequent apoptotic death cascade, and it reduced the generation of Aβ fragments by upregulating the activity of α-secretase ADAM10 and decreasing the activity of BACE1 via direct binding [242]. As mentioned previously, redox-active metal ions, such as copper and zinc, can bind to Aβ and stimulate its aggregation and production of ROS. In human neuroblastoma SK-N-BE(2)-M17 cells, myricetin was capable of modulating copper-induced aggregation of Aβ, probably by competing with Aβ for binding to copper ions. Moreover, it modulated metal-induced Aβ aggregation better than metal-free Aβ aggregation, demonstrating its specific reactivity toward metal-related Aβ species [243]. In silico studies revealed that myricetin, as well as quercetin, may inhibit cholinesterases via direct binding [192]. In rats with streptozotocin-induced neuronal damage, myricetin also promoted neuronal recovery and an increased number of intact neurons in hippocampal CA3 pyramidal layers, leading to cognitive improvements [244].

Likewise, in 1-methyl-4-phenylpyridinium (MPP^+^)-treated MES23.5 cells, a model for PD, myricetin reduced neuronal loss. It inhibited the production of ROS, preserved mitochondrial functioning and prevented apoptotic events by modulating mitogen-activated protein kinase kinase 4 (MKK4) and JNK activation [245]. Similar to the anti-amyloidogenic effect observed in AD, myricetin prevented aggregation of PD-related α-synuclein fibrils and destabilized preformed fibrils, indicating the non-specificity of its anti-fibrillogenic activity, which can be beneficial for various neurodegenerative diseases [246]. Furthermore, myricetin stimulates proteosomal degradation and elimination of abnormal proteins, thus suppressing aggregation of toxic proteins. It promotes degradation of misfolded proteins by enhancing endogenous levels of protein quality control components such as the molecular chaperone Hsp70, transcription factor HSF-1 and quality control ubiquitin ligase E6-AP. Elimination of protein aggregation has been demonstrated for mutant α-synuclein (related to PD), SOD1 (associated with amyotrophic lateral sclerosis) and polyglutamine-containing aggregates specific to HD [247].

Additionally, myricetin ameliorated brain injury and recovered motor, sensory and cognitive functions in a rat model of cerebral ischemia. It exerted neuroprotective (prevention of neuronal loss), anti-apoptotic (reduced number of TUNEL-positive cells) and anti-oxidative effects (amelioration of ROS and MDA production and increased activity of SOD and catalase). These effects were accompanied by an improvement in mitochondrial function, increased production of ATP and reduced mitochondrial oxidative damage. Myricetin triggered nuclear translocation of Nrf2 and enhanced the expression of HO-1, suggesting that the protection offered by myricetin was related to the Nrf2-induced mechanisms of the anti-oxidative defense [248]. The neuroprotective effects of myricetin are also related to inflammatory pathways as it may reduce the levels of NF-κB/p65, IL-1β, IL-6 and TNF-α. In addition, neuroprotective effects of myricetin could be achieved through activation of the Akt pathway and inactivation of the p38 pathway [249].

Finally, it should be mentioned that, in certain circumstances, myricetin, similar to quercetin, may exert a neurotoxic effect when applied together with excess copper. In SH-SY5Y cells, myricetin promoted the production of ROS and caspase-independent cell death without affecting p53 expression and its target Bax [197]. Hence, the adverse effects of quercetin, myricetin and potentially some other flavonols should not be neglected. Instead, further research is needed to obtain better insight into the context-dependent intracellular effects of different flavonols. On the other hand, direct evidence supporting the involvement of p53 in the neuroprotective effects of myricetin is still missing. Hence, research in the p53 field is an open window that could contribute to a further understanding of the neuroprotective potential of myricetin at the molecular level.

Laricitrin and syringetin are methylated forms of myricetin (O-methylated flavonols) that are, together with quercetin, myricetin and other flavonols, present in grapes and wine [250,251]. Although a neuroprotective potential of these flavonols is yet to be established, epidemiological studies suggest that moderate consumption of red wine, particularly in combination with the Mediterranean diet (which is also rich in flavonols and other flavonoids), may be a lifestyle factor with beneficial effects on the development and progression of neurodegenerative diseases based on the synergistic action of wine polyphenols and their ability to act together in a multi-target manner [252].

#### Dihydromyricetin

Dihydromyricetin (ampelopsin), structurally related to myricetin, is a flavanonol with anti-oxidative and anti-inflammatory effects. It has received great attention as a potential protective agent against neurodegenerative diseases. Thus, in PC12 cells, it prevented H_2_O_2_-induced production of ROS and neuronal apoptosis [253]. Antioxidant defense was mediated by ERK and Akt activation and increased expression of HO-1 [253]. Similarly, in HT22 hippocampal neurons, it inhibited OS and prevented apoptosis by activating the Nrf2/HO-1 pathway [254]. Dihydromyricetin also suppressed the brain aging of D-galactose-induced rats. It reduced the expression of miR-34a, attenuated astrocyte activation, inhibited apoptosis and improved autophagy by upregulating Sirt1 and downregulating mTOR pathways [255]. Together with the decreased expression of miR-34a and the increased activation of the Sirt1 pathway, dihydromyricetin alleviated the increase in p-p53 expression, suggesting that the effect of dihydromyricetin on the attenuation of neuronal death via the AMPK/Sirt1 pathway could be mediated via p53 inhibition [255]. Similarly, in an AD rat model induced by intracerebroventricular injection of Aβ42, dihydromyricetin prevented a drop in pAMPK and Sirt1 that ultimately downregulated the NF-κB pathway, and it improved learning and memory abilities. Action along the AMPK/Sirt1 signaling pathway attenuated the inflammatory response by reducing hippocampal levels of NF-κB p65, IL-1β, IL-6 and TNF-α and prevented apoptosis by increasing Bcl-2 and depleting Bax expression [256].

Dihydromyricetin also demonstrated promising potential for PD. In PC12 cells, it attenuated 6-OHDA-induced apoptosis, blocked ROS-induced activation of the p38 and JNK pathways and upregulated the Nrf2/ARE pathway by inhibiting the expression of GSK-3β [257]. Similarly, in an MPTP-induced mouse model of PD, dihydromyricetin was effective against motor impairments and prevented loss of dopaminergic neurons in the substantia nigra pars compacta by inhibiting the activity of GSK-3β [258]. It also prevented MPP^+^-induced generation of ROS in dopaminergic MES23.5 cells. In these cells, dihydromyricetin alone participated in Akt activation and increased the phosphorylation of GSK-3β at Ser9, thus inhibiting its activity. This inhibitory effect on GSK-3β activity was also evident during MPP^+^ exposure [258]. Interestingly, dihydromyricetin also exerts an antidepressant-like effect. Increasing evidence suggests that neuroinflammation and reduced levels of BDNF in the hippocampus are associated with depression-like behavior. By activating the ERK1/2-CREB pathway, and through phosphorylation of GSK-3β at Ser9, dihydromyricetin enhances BDNF expression in the hippocampus, suggesting that it also has potential as an antidepressant drug [259]. Myricetin also attenuated depressant-like behavior by normalizing BDNF levels in mice exposed to chronic stress, thus indicating another interesting route for the potential usage of flavonols in diverse brain and behavioral pathologies [260].

### 5.4. Myricitrin

Myricitrin is glycosylated form of myricetin, namely, the 3-O-rhamnoside of myricetin (Figure 5). It belongs to the flavonol group and is abundantly isolated from the root bark of *Myrica cerifera*. Its neuroprotective potential was reported in mice exposed to 6-OHDA. Myricitrin ameliorated loss of dopaminergic neurons, preserved the activity of tyrosine hydroxylase and suppressed the inflammatory response by attenuating TNF-α expression in activated microglia [261]. Its protective effects were also documented in traumatic injury of the spinal cord where myricitrin exerted strong anti-oxidative effects, reduced neuroinflammation, restored p53 expression and decreased the Bax/Bcl-2 ratio [262]. Regarding its anti-oxidative effects, myricitrin reversed the increase in MDA, as well as the activation of SOD, catalase and GSH-Px, whereas its anti-inflammatory effects were reflected in the inhibition of spinal cord injury-induced expression of the NF-κB p65 subunit, TNF-α, IL-1β and IL-6. Ultimately, its protective effects were observed at the histological level and in locomotor activity [262]. Although the neuroprotective effects of myricitrin have not been explored sufficiently, its anti-oxidative and anti-apoptotic effects have been described in other types of cells. For example, in H_2_O_2_-induced endothelial injury, myricitrin acted as an ROS scavenger and reduced the extent of lipid peroxidation. It decreased H_2_O_2_-induced apoptosis together with inhibition of p53 gene expression, activation of the caspase-3 and MAPK pathways and alteration in the expression of pro-apoptotic and anti-apoptotic genes [263].

### 5.5. Fisetin

Fisetin is a flavonol commonly found in strawberries. It interferes with the sumoylation of the p53 protein [264]. Covalent attachment of proteins from the small ubiquitin-related modifier (SUMO) family to target proteins is an important posttranslational modification that regulates a plethora of fundamental cellular processes, including the regulation of transcriptional activity, apoptosis and response to stress stimuli. Direct binding of fisetin to SUMO1 causes destabilization and unfolding of SUMO1 and prevents the sumoylation of p53 and, ultimately, its biological activity. Notably, molecular interactions of quercetin (in comparison with fisetin, which possesses one additional hydroxyl group at A5) and kaempferol (differs from fisetin in the position of one hydroxyl group) (Figure 5) were much weaker, suggesting a high specificity of fisetin–SUMO1 interactions [264]. Interestingly, in HT-22 cells (a model for oxidative glutamate toxicity that ends in GSH depletion), only fisetin and quercetin of several tested natural compounds were capable of attenuating the severe GSH drop. This was accompanied by a relatively low accumulation of ROS, probably because fisetin and quercetin maintained GSH levels below the threshold above which excessive generation of ROS is initiated [265]. Fisetin also promotes the differentiation of nerve cells, thus mimicking neurotrophic proteins. In particular, fisetin induced neurite outgrowth during differentiation of PC12 cells by activating ERK signaling [266].

The neuroprotective effects of fisetin against AD-related pathologies have been confirmed in vitro and in animal models. Thus, fisetin induced phosphorylation of CREB by activating ERK signaling, facilitated long-term potentiation in rat hippocampal slices and enhanced memory in mice [267]. Likewise, oral administration of fisetin to APPswe/PS1dE9 double transgenic AD mice from 3 to 12 months of age prevented memory problems. Preservation of memory functions correlated with increased activation of ERK and reduced OS and anti-inflammatory markers, and the maintenance of markers of synaptic function [268]. Fisetin-mediated activation of ERK also provided neuroprotection in several models of HD [269]. Akaishi and co-authors (2008) investigated structural requirements for the anti-amyloidogenic activity of fisetin. They tested 10 flavonoids, and among them, four flavonols, fisetin, luteolin, quercetin and myricetin, inhibited the formation of Aβ fibrils, whereas kaempferol, also a flavonol, promoted the formation of Aβ fibrils [270]. These findings indicate that the effects of flavonols cannot be generalized. The authors concluded that the 3’,4’-dihydroxyl groups of the B ring are essential for the inhibitory effect of luteolin on Aβ fibril formation, and that loss of the 3′- or 4′-hydroxyl group converts the effect from inhibitory to promoting. As myricetin was the most effective in the prevention of Aβ fibril formation, it is likely that increasing the number of hydroxyl groups, particularly on the B ring, results in a greater inhibitory effect [270]. Furthermore, fisetin largely reversed Aβ42-induced pathological changes in adult mice. It reduced BACE-1 expression and accumulation of Aβ and abrogated Aβ-induced suppression of PI3K/Akt/GSK-3β signaling. It was effective in preserving synaptic function and recovering memory functions. It also acted as an anti-inflammatory agent by suppressing the production of neuroinflammatory mediators p-IKKβ, p-NF-κB, TNFα and IL-1β and by reducing the number of activated glial cells, and as an anti-apoptotic agent by preventing activation of p53, cytochrome c, Bax and caspase-3, together with the upregulation of Bcl-2 in the hippocampus [271,272]. Neuroprotective effects of fisetin were also demonstrated in aluminum-induced Aβ aggregation. In the cortex and hippocampus of AlCl_3_-administered mice, fisetin reduced Aβ aggregation and the levels of p53, cytochrome c and caspase-9 and -3 and restored the Bax/Bcl-2 ratio, suggesting that it could be effective in slowing down or preventing AD [273].

Regarding PD, in a mouse model of MPTP-induced toxicity, fisetin reduced behavioral deficits and prevented the depletion of tyrosine hydroxylase-positive cells in the substantia nigra [274]. In cellular models exposed to MPTP, it also improved viability, reduced α-synuclein levels and prevented the depletion of dopaminergic markers [275,276].

Furthermore, fisetin rescued mouse brains from synaptic and cognitive dysfunctions, OS and neuroinflammation induced by D-galactose. D-galactose induces brain aging and is commonly used for studying neuroprotective strategies against age-related neurological diseases. By activating the Sirt1/Nrf2 pathway, fisetin partially prevented the accumulation of ROS and reduced the levels of lipid peroxidation that further suppressed the inflammatory response via the pJNK/NF-κB pathway. This reduced downstream production of inflammatory mediators such as IL-1β and TNF-α, and activation of glial cells. Moreover, the expression of D-galactose-induced apoptotic markers was reduced by fisetin [277,278]. Additionally, fisetin ameliorated mitochondrial membrane depolarization, induced the expression of autophagy-related genes, reversed the D-galactose-induced decrease in the activities of synaptosomal membrane transporters Na^+^/K^+^-ATPase and Ca^2+^- ATPase and stimulated acetylcholinesterase activity [277].

### 5.6. Kaempferol

Kaempferol is yet another flavonoid from the flavonol group abundantly present in food and beverages. It is considered as a safe and efficient anti-inflammatory agent, although caution is required as some in vitro studies have demonstrated its carcinogenic and toxic properties [279,280]. Regarding its neuroprotective potential, it protected PC12 cells from the toxic effects of Aβ [281], and SH-SY5Y cells and primary neurons from rotenone-induced toxicity [168,282]. In PC12 cells, kaempferol also attenuated HNE-induced activation of the JNK pathway and apoptotic events by directly inhibiting NADPH oxidase [283]. In rotenone-treated cells, kaempferol counteracted the increase in ROS production, rescued mitochondrial function and prevented the apoptotic cascade. The authors suggested enhancement of mitophagy as the main mechanism involved in the anti-oxidative and anti-apoptotic effects of kaempferol, based on the increased mitochondrial fission rate and increased number of mitochondria-containing autophagosomes [168]. In the transgenic *Drosophila* model of PD, kaempferol was also effective as an ROS scavenger. It reduced OS markers (TBARS and protein carbonyl content, activity of monoamine oxidase), increased the activity of glutathione-S-transferase, catalase and SOD, reduced caspase-3 and -9 activity and preserved the expression of tyrosine hydroxylase. It also delayed the loss of climbing activity and improved rewarding, dopamine-related reproductive behavior and cognitive functions in PD flies. Studies of ligand–protein interactions have shown that kaempferol is capable of binding to human α-synuclein, likely indicating its possibility to inhibit α-synuclein aggregation [284]. The neuroprotective effects of kaempferol were confirmed in an MPTP-induced mouse model of PD, where kaempferol improved striatal dopamine levels, increased the number of dopaminergic cells and improved behavioral deficits, likely by raising the anti-oxidative capacity, as evidenced by the restoration of SOD and GSH-Px activity, and the reduced MDA content [285]. Similar neuroprotective effects of kaempferol were demonstrated in a rotenone-induced rat model of PD. Besides its anti-oxidative and anti-apoptotic effects, preservation of tyrosine hydroxylase activity and improvement in dopamine levels and behavioral deficits, kaempferol inhibited the production of pro-inflammatory cytokines IL-6 and TNF-α, suggesting that its neuroprotective ability relies, at least partially, on the NF-κB pathway [282].

Furthermore, protective effects of kaempferol have been demonstrated in a transgenic *Drosophila* model of AD [286]. Flies fed on a diet enriched with kaempferol similarly showed a delay in memory loss and, in comparison to Aβ flies, had a higher GSH content, reduced lipid peroxidation and protein carbonyl content, decreased activity of caspase-3 and -9 and increased acetylcholinesterase activity [286]. Kaempferol also protected mice from D-galactose-induced cognitive impairments, ameliorated OS and stimulated activation of the ERK/CREB pathway [287]. Furthermore, it enhanced Na^+^/K^+^-ATPase activity. As Na^+^/K^+^-ATPase is crucial for maintaining Na^+^/K^+^ gradients, regulation of its function is important for proper neuronal functioning. Regarding p53 and kaempferol, studies in neuronal cells are still missing. However, its hepato-protective effects in rats were associated with the activation and upregulation of nuclear levels of Sirt1, and the subsequent prevention of nuclear acetylation of p53 and NF-κB p65 [288]. Similar effects, together with the reduced acetylation of the Nrf2 pathway, were observed in retinal pigment epithelial cells exposed to H_2_O_2_ [289], altogether suggesting that Sirt1/Nrf2/p53 signaling could be activated in kaempferol-treated neuronal cells in OS conditions.

### 5.7. Morin

Morin, originally isolated from the members of the *Moraceae* family, is a flavonol present in many fruits and herbs, and red wine [290,291]. Regarding its potential in ameliorating pathological changes in AD, morin attenuated Aβ-induced overproduction of ROS, activated endogenous mechanisms of the anti-oxidative enzymatic defense, mitigated protein carbonylation and prevented OS in cultured cortical neurons and organotypic slices. These anti-oxidative effects preserved mitochondrial function, ATP synthesis and energetic homeostasis, inhibited cytochrome c release and prevented neuronal injury and death [291]. In APPswe/PS1dE9 double transgenic mice, morin prevented cognitive deficits by reducing BACE1 activity, APP processing via the amyloidogenic pathway and plaque burden, and by stimulating degradation of Aβ. This reduced the activation of microglial cells and increased the expression of synaptic proteins [292]. In addition, it reduced tau hyperphosphorylation by inhibiting cdk5, whereas in a triple transgenic AD mouse model, it attenuated hyperphosphorylation of tau by inhibiting the activity of GSK-3β [292,293].

The neuroprotective potential of morin against pathological changes characteristic of PD has also been investigated. In MPP^+^-induced apoptosis in differentiated PC12 cells, morin prevented ROS production and neuronal apoptosis by preventing an increase in caspase-3 activity, whereas in MPTP-exposed mice, it attenuated the levels of TNF-α, motor behavioral deficits, striatal dopamine depletion and loss of dopaminergic neurons [290,294]. Furthermore, it protected primary neurons from the MPP^+^-induced increase in ROS production and disruption of the mitochondrial membrane potential, prevented translocation of NF-κB in cultured astrocytes and attenuated astrocyte activation and production of TNF-α [294].

Moreover, it has been shown that morin may prevent neurotoxicity induced by ifosfamide, a widely prescribed chemotherapeutic drug. In rats exposed to ifosfamide, morin inhibited acetylcholinesterase activity, reduced lipid peroxidation, prevented depletion of the GSH pool and stimulated the activity of Nrf2 and antioxidant enzymes. Moreover, it downregulated apoptotic (p53, caspase-3, JNK pathway) and inflammatory (NF-κB, nNOS, TNF-α) markers, demonstrating its ability to suppress OS, apoptosis and neuroinflammation in the brain, altogether preventing histopathological changes in the hippocampus and suggesting its great potential in alleviating the neurotoxic effects of chemotherapy, but also its potential in the therapy of OS-related neurodegenerative diseases [295]. Similar protective effects of morin have been observed against doxorubicin-induced cognitive impairment [296], in streptozotocin-induced brain changes of diabetic rats [297] and against diabetic neuropathy, a chronic pain condition that is accompanied by significantly increased production of ROS and pro-inflammatory cytokines in peripheral neurons [298].

### 5.8. Isorhamnetin

Isorhamnetin, a 3’-O-methylated derivative of quercetin (Figure 5), is one of the most important bioactive components from the leaves of *Ginkgo biloba* L. Among other pharmacological activities, it offers protection against cerebrovascular diseases and is capable of exerting anti-oxidative, anti-apoptotic and anti-inflammatory effects by modulating signaling pathways, including the PI3K/Akt, MAPK, Nrf2 and NF-κB pathways, in various pathological conditions and model systems [299,300]. In PC12 cells challenged with H_2_O_2_, isorhamnetin improved viability, reduced the intracellular formation of ROS, increased catalase activity and prevented the activation of caspase-3. It also stimulated Akt activation and modulated the p38 and JNK pathways, emphasizing its role as a signaling molecule [301]. Furthermore, isorhamnetin alleviated endoplasmic reticulum stress-induced apoptosis in N2a cells by inhibiting cytosolic Ca^2+^ overload, ROS production and apoptotic events, at least in part, by promoting phosphorylation of the protein kinase PKCε [302], whereas in HT22 hippocampal neurons exposed to high glucose-aggravated oxygen–glucose deprivation and reoxygenation, it attenuated apoptosis, the inflammatory response and OS via the Akt/Sirt1/Nrf2/HO-1 signaling pathway [303]. It also promoted functional recovery after spinal cord injury in rats by activating the Nrf2/HO-1 pathway, and by suppressing microglial activation and production of inflammatory cytokines [304]. Similar anti-oxidative and anti-inflammatory effects were observed against ischemic injury in mice [305]. Moreover, in PC12 cells, isorhamnetin promoted neuronal differentiation and robustly induced the expression of neurofilaments by potentiating the effects of nerve growth factor (NGF) on neurite outgrowth [306]. Regarding AD pathology, in human neuroblastoma SH-SY5Y cells, isorhamnetin improved viability, prevented morphological changes in Aβ-treated cells and inhibited aggregation of Aβ, probably by destabilizing Aβ fibrils [307]. In Aβ25-35-induced memory impairment and oxidative damage in rats, it increased the activity of acetylcholinesterase, attenuated the increase in MAO activity and reduced H_2_O_2_ production and expression of iNOS and IL-β mRNA in the hippocampus [308]. It also prevented scopolamine-induced learning and memory deficits in mice. At the cellular and molecular levels, the beneficial effects of isorhamnetin on cognitive functioning were associated with anti-oxidative effects evidenced by increased GSH levels, increased SOD and catalase activity and reduced MDA generation. In the same study, it was shown that isorhamnetin reversed scopolamine-induced nitrosative stress, increased acetylcholinesterase activity and enhanced BDNF levels in the prefrontal cortex and hippocampus, indicating its ability to enhance synaptic plasticity [309]. Stimulatory effects of isorhamnetin on cholinergic neurotransmission could also be achieved through the inhibition of acetylcholinesterase activity [310]. All these findings demonstrate that isorhamnetin could be effective in the early management of neurodegenerative diseases, which needs to be explored further in animal models and clinical studies. In several cellular models, it exerted toxic effects, and thus its toxicological profile should be carefully examined [300].

### 5.9. Rhamnazin

Rhamnazin is yet another methylated derivate of quercetin (Figure 5). Its anti-oxidative (free radical scavenging) and anti-inflammatory properties, together with its activation of the Nrf2 pathway, have been observed in vitro and in non-neuronal tissue [311]. Furthermore, rhamnazin inhibits the secretory phospholipase A2, which is one of the major targets in the development of anti-inflammatory drugs and is upregulated in AD brains [312,313], and can act as an acetylcholinesterase inhibitor [314]. Although the neuroprotective potential of rhamnazin in neurodegenerative conditions has yet to be explored, it has been shown that it may prevent repeated restraint stress-induced cognitive impairment in rats, at least partially, by increasing BDNF levels in the hippocampus [315] and ameliorate TBI in mice by reducing OS, neuroinflammation and apoptosis [316].

### 5.10. Azaleatin

Azaleatin (quercetin 5-methyl ether) is a flavonol whose antioxidant activity has been confirmed in vitro. It scavenged 2,2-diphenyl-1-picrylhydrazyl (DPPH) radicals and superoxide radicals and showed reducing power capacity, although metal chelating activity was not observed [317]. In silico analysis revealed that it can act as an inhibitor of acetylcholinesterase activity [314], but its neuroprotective properties, ability to inhibit protein aggregation and modulatory effects on signal transduction are yet to be studied in cellular and animal models.

### 5.11. Gossypetin

Gossypetin is a flavonol found in plants from *Hibiscus* species. According to in silico analysis, gossypetin may inhibit acetylcholinesterase activity [314]. Radical scavenging activities have also been documented [311], as well as the ability to prevent heparin-induced assembly of tau filaments in vitro [318]. Modulation of the Nrf2, NF-κB and p53 pathways has been observed, together with anti-oxidative, anti-inflammatory and anti-apoptotic effects [319,320], albeit not in models of neurodegenerative diseases. Thus, current findings indicate its therapeutic potential, but further studies are needed.

## 6. Conclusions

The therapy of neurodegenerative diseases is still a source of frustration for a myriad of patients, doctors and researchers. The need for disease-modifying medications capable of altering the course of these devastating conditions is urgent. It is generally accepted that effective therapeutics must target several aspects of neurodegenerative diseases at a time, as pathological changes disrupt the functioning of multiple cellular systems due to their complex nature and mutual interplay.

**Figure 7:**
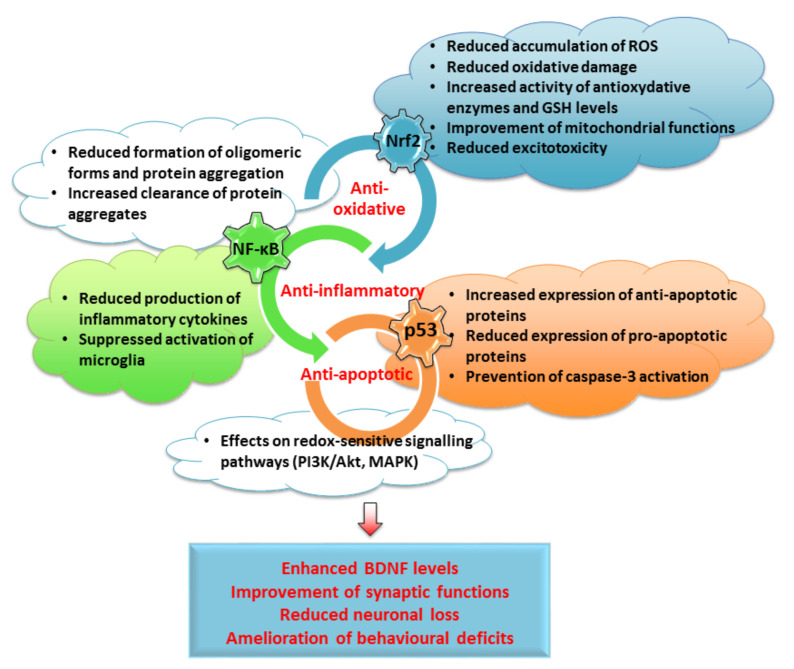
Molecular and cellular mechanisms underlying the anti-oxidative, anti-inflammatory and anti-apoptotic effects of flavonols.

Flavonols are appreciated as a relatively safe and non-toxic therapeutic option against neurodegenerative diseases. In various in vitro and in vivo models, they have demonstrated desirable neuroprotective effects and multiple biological activities based on various mechanisms of action at the molecular and cellular levels. Mounting evidence indicates that they may suppress the severity of the major molecular hallmarks of neurodegenerative processes: OS, neuroinflammation and apoptotic death, and alleviate behavioral deficits (Figure 7 and Table 1).

Although, at first, they were admired as powerful antioxidants, due to their low bioavailability and problematic penetration across the BBB, they are found in the brain at very low amounts, even after prolonged periods of supplementation. Hence, it is unlikely that in such small concentrations they would outcompete the action of abundantly present endogenous antioxidants. It is more likely that they exert their beneficial effects by acting on different components of signaling cascades, of which those related to OS, neuroinflammation and apoptotic death, such as Nrf2, NF-κB and p53, are particularly important. The observed modulatory and regulatory effects of flavonols on the expression and activity of these transcriptional factors anticipate their potential in alleviating pathological changes accompanying neurodegenerative diseases. However, further studies are needed to better characterize their effects, particularly in environments with disturbed metal homeostasis. On the other hand, although the results from in vitro and in vivo studies are promising, clinical trials are still missing and must be carried out to reveal the real potential of flavonols in clinical settings.

Research directed towards their better absorption and enhancement of their metabolic stability must be performed in parallel with pharmacological studies. Nanotechnology-supported interventions are offering a great opportunity to overcome the problem of successful delivery, increasing the chance that oral consumption of flavonols could be a promising approach in alleviating the progression of pathological changes in neurodegenerative diseases. In medicinal chemistry, flavonols are appreciated as good drug precursors and a valuable template for the development of more effective therapeutics, which makes them promising candidates in the development of novel neuroprotective strategies.

## Figures and Tables

**Table 1:** Anti-oxidative, anti-inflammatory and anti-apoptotic effects of flavonols in various experimental conditions related to neurodegenerative diseases.

Model System	Treatment	Anti-Oxidative Effects	Anti-Inflammatory Effects	Anti-Apoptotic Effects	Reference
Quercetin					
PC12 cells	H_2_O_2_	↓ ROS production, ↓ MDA levels, ↑ GSH content, ↑ catalase, SOD and GSH-Px expression		↓ Bax and ↑ Bcl-2 expression, ↓ cleaved caspase-3 and -9, ↓ p53 expression, ↑ activation of PI3K/Akt	[203,204,206]
PC12 cells	Aβ25-35	↓ levels of MDA, and HO-1 protein, ↑ levels of SOD, GSH-Px, catalase, Sirt1 and Nrf2 protein			[212]
P19 neurons	H_2_O_2_	↓ ROS production		↓ caspase-3/7 activation, ↓ nuclear condensation, ↓ p53 and ↑ Bcl-2 expression, ↑ activation of PI3K/Akt and ERK1/2	[186,188]
Cerebellar granule neurons	H_2_O_2_	↑ GSH levels, Nrf2 translocation, ↑ expression of GCLC mRNA			[205]
P19 neurons	CuSO_4_	↓ ROS production in moderate OS, pro-oxidative activity in severe OS		↓ caspase-3/7 activation, ↓ chromatin condensation, ↓ PUMA expression, ↑ activation of PI3K/Akt and ERK1/2	[123]
HT22 cells	Okadaic acid	↓ levels of ROS and MDA, ↑ levels of SOD and GSH-Px, ↑ mitochondrial membrane potential	↓ expression of NF-κB p-p65	↓ Bax levels, ↓ cleaved caspase-3, activation of PI3K/Akt pathway, inhibition of p38 and JNK, reduced intracellular Ca^2+^ levels	[213,215]
*Drosophila* AD model				Restored expression of genes involved in p53 signaling	[219]
Wild-type C57BL/6N mice	LPS		↓ gliosis, ↓ expression of p-NF-κB, ↓ levels of TNF-α, IL-1β, COX-2 and iNOS	↓ cytochrome c release and Bax/Bcl-2 ratio, ↓ caspase-3 activity, ↓ synaptic loss	[200]
Wistar albino rats (hippocampus)	Al-lactate	↓ ROS production, ↑ MnSOD activity, preserved mitochondrial morphology		↓ Bax and ↑ Bcl-2 expression, ↓ cytochrome c expression, ↓ caspase-3 activation, ↓ DNA fragmentation, ↓ p53 expression	[207]
APPswe/ PS1dE9 mice (C57/BL) (cortex, hippocampus)		Restoration of mitochondrial membrane potential, ROS levels and ATP production			[209]
3xTg-AD mice (hippocampus)			↓ activation of microglia, ↓ iNOS and COX-2 immunoreactivity, ↓ IL-1β		[210]
Sprague Dawley rats (brain homogenate)	Aβ	↑ SOD, catalase and GSH, ↓ MDA, activation of Nrf2/HO-1 pathway			[220]
MN9D dopaminergic cells		↑ mitochondrial biogenesis and mtDNA content		↑ pAkt expression, ↑ BDNF levels	[225]
SK-N-MC neuroblastoma cells	Mn	↑ SOD and catalase activity, ↑ GSH levels, ↓ MDA, ↑ levels of Nrf2 and HO-1	↓ level of P-IκBα and NF-κB p65	↓ staining with Hoechst 33342, ↓ number of apoptotic cells	[226]
Albino rats	rotenone	↓ MDA levels, ↑ thioredoxin reductase reactivity		↓ DNA fragmentation	[222]
Sprague Dawley rats (brain homogenate)	Mn	↓ ROS and protein carbonyl levels, ↑ Cu/Zn-SOD activity, ↑ Nrf2 and HO-1 mRNAs	↓ TNF-α, IL-1β, IL-6, COX-2 and iNOS protein expression, ↓ NF-κB and iNOS mRNAs	↓ Bax expression, ↓ cytochrome c expression, ↓ cleaved caspase-3 expression, ↓ PARP-1 levels	[226]
3-NP-induced model of HD in Wistar rats (striatum)		Restored ATP levels, ↑ mitochondrial SOD and catalase activity, ↓ MDA levels and ↑ thiol content		↓ number of pyknotic and condensed nuclei	[227]
Rutin					
Sprague Dawley rats (hippocampus)	TMT		↓ mRNA markers of reactive microglia and pro-inflammatory cytokines		[234]
Wistar rats (hippocampus)	STZ	↓ TBARS content and nitrite levels, ↑ GSH, ↑ GPx, GR and catalase activity	↓ GFAP, COX-2, iNOS, IL-8 and p65 NF-κB expression	Restored PARP-1 activity, prevented histological abnormalities	[235]
PP/PS1 (APP_swe_/ PSEN1dE9) mice (brain homogenate)			↓ microglial activation, ↓ TNF-α, IL-1β, IL-6, ↑ IL-4 and IL-10	Reversed synaptic dysfunction, enhanced microglial Aβ phagocytosis	[236]
Sprague Dawley rats (brain homogenate)	Colistin	↑ SOD, GSH-Px and catalase content, ↑ GSH levels, ↓ MDA, ↑ levels of Nrf2	↓ GFAP expression, ↓ expression of NF-κB, TNF-α, ↓ nNOS activity	↑ Bcl-2 and ↓ Bax expression, ↓ caspase-3 activity, ↓ p53 level, modulation of ERK1/2 pathway	[238]
Myricetin					
Primary neuronal cultures	Glutamate	↓ ROS production		↓ calcium overload, ↓ activation of caspase-3, ↓ nuclear fragmentation	[241]
Primary neuronal cultures	Aβ			↓ nuclear fragmentation, ↓ caspase-3 activation	[242]
MES23.5 cells	MPP^+^	↓ ROS production, restored mitochondrial membrane potential		↓ nuclear condensation, ↓ Bax/Bcl-2 ratio, ↓ caspase-3 activation, ↓ pJNK expression	[245]
Myricitrin					
C57BL/6 mice (substantia nigra)	6-OHDA		↓ number of Iba1-positive cells, ↓ expression TNF-α		[261]
Sprague Dawley rats (brain homogenate)	TBI of the spinal cord	↑ SOD, GSH-Px and catalase content, ↓ MDA levels	↓ TNF-α, IL-1β, IL-6 and NF-κB p65 subunit content, ↓ COX-2 and TGF-β1 mRNA expression	↓ Bax/Bcl-2 ratio, ↓ p53 levels	[262]
Fisetin					
HT22 cells	Glutamate	↓ ROS production, ↑ GSH levels			[265]
APPswe/ PS1dE9 mice (hippocampus)		↓ protein carbonylation	↓ cPLA2 levels, ↓ COX-2, ↓ astrocytic reactivity	↑ pERK, preserved expression of synaptic markers	[268]
C57BL/6N mice (hippocampus)	Aβ		↓ expression of p-IKKβ, p-NF-ҡB, TNFα and IL-1β, ↓ number of activated Iba-1- and GFAP-positive cells	↓ Bax expression, ↑ Bcl-2 expression, ↓ cytochrome c activation, ↓ cleaved caspase-3 and -9, ↓ expression of p53 and PARP-1, ↑ activation of PI3K/Akt, restored synaptic function	[271]
C57BL/6N mice (hippocampus)	LPS	↓ ROS production and lipid peroxidation, ↑ GSH levels	↓ reactivity of TLR4 and pNF-ҡB, ↓ TNFα, IL-1β and COX-2, ↓ activation of Iba-1- and GFAP-positive cells	↓ expression of cytochrome c, Apaf-1, caspase-3 and -9 and PARP-1, ↓ activation of JNK pathway, prevented morphological changes	[272]
Swiss albino mice (cortex and hippocampus)	AlCl_3_			↓ Bax and ↑ Bcl-2 mRNA expression, ↓ expression of cytochrome c, caspase-3 and -9, ↓ expression of p53, ↓ number of TUNEL-positive neurons, ↓ JNK activation	[273]
C57BL/6N mice (striatum)	MPTP			↓ number of TUNEL-positive neurons	[274]
Wistar rats (brain homogenate)	D-galactose	↓ production of ROS and NO, ↓ protein carbonylation and lipid hydroperoxidation, ↑ SOD and catalase levels, ↑ total thiol content	↓ expression of TNF-α and IL-1β		[277]
C57BL/6N mice	D-galactose	↓ ROS and MDA levels, ↑ levels of Sirt1, Nrf2 and HO-1	↓ pNF-ҡB, NF-ҡB, Iba-1, GFAP, IL-1β, TNF-α and iNOS	↓ Bax and ↑ Bcl-2 expression, ↓ expression of caspase-3 and PARP-1	[278]
Kaempferol					
PC12	HNE			↑ Bcl-2 levels, ↓ caspase-3 activation, ↓ nuclear condensation and PARP-1 cleavage, inhibited activation of NADPH oxidase and JNK phosphorylation	[283]
SH-SY5Y cells	Rotenone	↓ production of ROS, ↓ mitochondrial carbonyls, restored mitochondrial function		Prevented round-shape phenotype, preserved nuclear morphology, ↓ reduced caspase-9/3 cleavage, ↓ activation of JNK and p38	[168]
SH-SY5Y cells	Rotenone	↓ production of ROS, ↓ MDA content, ↑ GSH, SOD, GSH-Px and catalase levels		↑ expression of TH	[282]
Albino rats	Rotenone	↓ production of ROS, ↓ MDA levels, ↑ GSH, SOD, GSH-Px and catalase levels	↓ levels of TNF-α and IL-6		[282]
*Drosophila* model of PD		↑ free radical scavenging, ↓ TBARS and protein carbonyl content, ↑ SOD, GSH-S-transferase and catalase activity		↓ reduced caspase-3 and -9 activity, ↑ TH staining and L-DOPA levels	[283]
*Drosophila* model of AD		↑ GSH content, ↓ TBARS and protein carbonyl content		↓ caspase-3 and -9 activity	[286]
C57BL/6 mice (substantia nigra)	MPTP	↑ SOD and GSH-Px activity, ↓ MDA content		↑ TH staining	[285]
Kunming mice (hippocampus)	D-galactose	↓ TBARS, ↑ SOD		↑ activation of ERK/CREB pathway	[287]
Morin					
SH-SY5Y cells	Aβ	↓ production of ROS		↓ staining with Hoechst 33342 and PI	[293]
Differentiated PC12 cells	MPP^+^	↓ production of ROS		↓ caspase-3 activity	[290]
Primary cultured neurons	MPP^+^	↓ ROS production, prevented mitochondrial membrane disruption		↓ staining with Hoechst 33342 and PI	[294]
Primary cultured astrocytes	MPP^+^		↓ astrocyte activation, ↓ nuclear translocation of p65 NF-ҡB		[294]
Cultured cortical neurons	Aβ	↓ ROS production and protein oxidation, ↑ SOD and catalase activity, ↓ calcium uptake and mitochondrial membrane depolarization, restored respiratory capacity		↓ PI staining, ↓ cytochrome c release	[291]
Sprague Dawley rats (brain homogenate)	Ifosfamide	↓ MDA levels, ↑ GSH, SOD, GSH-Px and catalase activities, ↑ Nrf2 levels	↓ GFAP levels, ↓ NF-ҡB and TNF-α, ↓ nNOS activity	↓ caspase-3 and p53 levels, ↓ JNK activation	[295]
APPswe/ PS1dE9 mice (cortex, hippocampus)			↓ activation of glial cells	↓ cerebral Aβ production, ↑ expression of synaptic markers	[291]
C57BL/6 mice (striatum and substantia nigra)	MPTP		↓ astrocyte activation, ↓ levels of TNF-α	↓ loss of dopaminergic cells	[294]
B57/BL mice (substantia nigra)	MPTP			↑ number of TH-positive cells	[290]
Isorhamnetin					
PC12 cells	H_2_O_2_	↓ formation of ROS, ↑ catalase activity		↓ calcium levels, ↓ caspase-3 activation, ↑ Akt signaling and ↓ activation of JNK pathway	[301]
N2a cells	TG	↓ formation of ROS		↓ calcium overload, ↓ PI fluorescence, ↑ phosphorylation of PKCε	[302]
HT22 neurons	OGD/R	↑ Akt/Sirt1/Nrf2/HO-1 pathway			[303]
Albino rats (hippocampus)	Aβ25-35	↓ formation of H_2_O_2_, ↓ MAO activity	↓ expression of iNOS and IL-1β mRNA		[308]
Albino mice (prefrontal cortex and hippocampus)	SCP	↓ MDA and nitrite production, ↑ GSH levels, ↑ SOD and catalase activities			[309]

Aβ—amyloid β; cPLA2—cytosolic phospholipase A2; GSH—glutathione; GCLC—glutamate–cysteine ligase catalytic subunit; GFAP—glial fibrillary acidic protein; GSH-Px—glutathione peroxidase; MAO—monoamino oxidase; MDA—malondialdehyde; MPP^+^—1-methyl-4-phenylpyridinium; MPTP—1-methyl-4-phenyl-1,2,3,6-tetrahydropyridine; 3-NP—3-nitropropionic acid; OGD/R– oxygen–glucose deprivation and reoxygenation; 6-OHDA—6-hydroxydopamine; PI—propidium iodide; PUMA—p53-upregulated mediator of apoptosis; ROS—reactive oxygen species; SCP—scopolamine (muscarinic antagonist); SOD—superoxide dismutase; STZ—streptozotocin; TG—thapsigargin (inductor of endoplasmic reticulum stress); TBARS—thiobarbituric acid reactive substances; TH—tyrosine hydroxylase; TLR4—Toll-like receptor 4; TMT—trimethyltin.
